# UPLC-QTOF-MS with a chemical profiling approach for holistic quality evaluation between a material reference of Wen Dan decoction and its commercial preparations

**DOI:** 10.1186/s13020-023-00767-z

**Published:** 2023-05-30

**Authors:** Siyu Yang, Gan Chen, Man Yuan, Yan Zou, Hongmei Zhang, Hongxi Xu

**Affiliations:** 1 School of Pharmacy, Shanghai University of Traditional Chinese Medicine, Shanghai, 201203 People’s Republic of China; 2 Engineering Research Center of Shanghai Colleges for TCM New Drug Discovery, Shanghai, 201203 People’s Republic of China; 3 Shineway Pharmaceutical Group Ltd., Hebei, China; 4 Shuguang Hospital, Shanghai University of Traditional Chinese Medicine, Shanghai, 201203 People’s Republic of China

**Keywords:** Wen Dan decoction, Material reference, Commercial preparations, UPLC-QTOF-MS, Holistic quality evaluation

## Abstract

**Background:**

Wen Dan decoction (WDD) has been a famous classic formula for resolving phlegm since ancient times in China. Currently, there are many types of WDD commercial preparations produced through modern technology. However, it is not known whether the holistic quality of WDD commercial preparations is consistent with the traditional decocting method to exert its proper effects. Therefore, the WDD material reference was studied and prepared, which can represent the traditional Chinese formulation WDD.

**Methods:**

A method based on UPLC-QTOF-MS was developed to evaluate the quality of WDD material reference and commercial prescriptions. At the same time, the multivariate statistical method was used to compare the differences between the material reference and the commercial prescription by principal component analysis (PCA) and heatmap. Finally, the UPLC-QTOF-MS method was established to quantitatively study 11 representative components, including naringin, hesperidin, neohesperidin, liquiritin, glycyrrhizic acid, adenosine, liquiritigenin, tangeretin, eriocitrin, naringenin and synephrine.

**Results:**

A total of 107 compounds were identified in the WDD material reference by comparing the retention time and fragment ion characteristics, including 54 flavonoids, 14 triterpenes, 10 organic acids, 7 alkaloids, 7 coumarins and 15 other components. The samples were almost evenly split into two groups, indicating a difference in quality between the WDD material reference and its commercial preparations in multivariate statistical analysis. Eleven major components of linearity, precision, repeatability, stability and recovery rate met the requirements, which were clearly different in commercial preparations and WDD material references. In terms of the content of 11 components in the commercial preparation, only CP8 is close to the material reference, which is in agreement with the statistical analysis of the heatmap. The concentrations of naringin and neohesperidin from the WDD material reference were higher than those from the commercial preparations.

**Conclusions:**

The quality evaluation method established in this study can be used to identify different sources of WDD but also proves that the WDD material reference contains higher naringin. Furthermore, this study confirmed that the preparation technology of WDD commercial prescriptions should be optimized on the basis of WDD material references, producing the closest possible clinical basis for the substance.

**Graphical Abstract:**

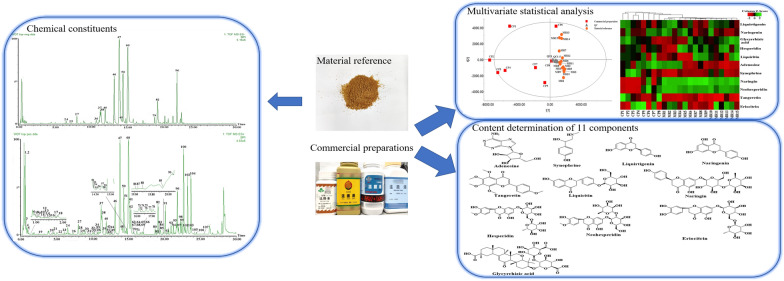

**Supplementary Information:**

The online version contains supplementary material available at 10.1186/s13020-023-00767-z.

## Introduction

The famous classical formula possesses complete illness preventive and treatment methods with a 3000-year history and a wealth of important experience in disease treatment. The integrated and synergistic actions on various targets of traditional Chinese medicine (TCM) have received much recognition [1]. Wen Dan decoction (WDD), which comprises 6 Chinese medicines, Pinelliae Rhizoma, Bambusae Caulis in Taenias, Aurantii Fructus Immaturus, Citri Reticulatae Pericarpium, Glycyrrhizae Radix et Rhizoma, and Zingiberis Rhizoma with a ratio of 2:2:2:3:1:4, is a famous classical Chinese formula. The National Administration of Traditional Chinese Medicine in China first issued the “One hundred Ancient Famous Classical Chinese Medicine Formula Catalogue”, which contained WDD and served as the foundation for its development [2, 3]. WDD was mentioned in Emergent Prescriptions Worth Thousands of Gold, a clinical TCM treatise authored by Sun Simiao during the Tang Dynasty. According to the requirements of the Famous Classical Formula, it can only provide pharmaceutical studies and nonclinical safety studies and may not provide efficacy and clinical studies. However, these studies must refer to ancient medical records for research to ensure the effectiveness and safety of the formulas. Material references are known as Chinese medicinal substances that exert therapeutic effects in the clinic based on the preparation of ancient famous classical Chinese medicinal formulas. Therefore, quality evaluation and control studies of material references would play an important role in the research and development of classic famous prescription drugs. The plant origin of medicine, processing, and decocting methods were used to determine the material reference technology of WDD. Presently, there are some commercially available WDD preparations. The safety and effectiveness of WDD commercial preparations in the clinic have not been reported thus far. Chinese formulas have complex components, and most have medicinal effects as a whole. The material reference serves as a quality benchmark for the ancient famous classical Chinese medicinal formulas and a standard reference for measuring Chinese medicine granules and commercial preparations. Therefore, this is a critical concern regarding the effectiveness equivalency of WDD material references and their commercial medications.

Currently, most of the reports on WDD as well as its addition and subtraction are focused on clinical application and pharmacological effects. There are few studies on holistic quality control and the differences between WDD material references and their commercial preparations, and they mainly concentrate on fingerprint research [4, 5] and content determination of representative components [6, 7]. In recent years, UPLC-QTOF-MS has been widely used in a variety of sectors. According to the fragment information of the compound under high energy, UNIFI software may offer the probable cracking approach for detected compounds under the given filter parameters [8]. An integrated project based on UPLC-QTOF-MS paired with the UNIFI informatics platform was used to disclose the chemical profile of WDD. In addition, the major representative components were simultaneously quantified. It is widely acknowledged that TCMs produce efficacy via multiple components on several targets. The key markers of the difference between the Famous Classical Chinese Formula and its commercial variants may be revealed using multivariate statistical analysis. In this study, the UPLC-QTOF-MS method was used to identify and quantify some important components, and multivariate statistical analysis was carried out on the WDD material reference and its commercial preparations. This also serves as a significant reference for future study and improvement of this formula, allowing researchers and clinicians to make a better choice for the source of WDD.

## Materials and methods

### Chemical reagents and materials

The reference compounds naringin (lot No. H24N9Z75896), hesperidin (lot No. M02J9S64781), neohesperidin (lot No. G10S11L123540), liquiritin (lot No. Z10J8X39611), glycyrrhizic acid (lot No. P13A9L67602), adenosine (lot No. N24011W135689), liquiritigenin (lot No. P2285834), tangeretin (lot No. H24F11K108893), eriocitrin (lot No. F27GB140285), naringenin (lot No. L21O10Q100513) and synephrine (lot No. Y26O7Y17088) were obtained from Shanghai Yuanye Biotechnology Co., Ltd. (Shanghai, China). The purities of the 11 reference compounds were all above 98%. Distilled deionized water was obtained using Milli-Q water purification equipment from Millipore (Bedford, MA, USA). MS-grade methanol and acetonitrile were purchased from Merck Company (Darmstadt, Germany). Formic acid (MS grade) was purchased from Fisher Scientific (Pittsburgh, USA).

WDD was prepared from 6 Chinese medicines according to the original formula in “Emergent Prescriptions Worth Thousands of Gold”. The processing method of each medicinal material was determined by ancient books and optimization experiments. Pinelliae Rhizoma was washed in hot water, and Aurantii Fructus Immaturus and Glycyrrhizae Radix et Rhizoma were first soaked and then stir-fried. Bambusae Caulis in Taenias, Citri Reticulatae Pericarpium and Zingiberis Rhizoma Recens were cleaned and sorted. All abovementioned medicines were identified by associate Professor Hongmei Zhang based on the monographs of each Chinese medicine documented in the China Pharmacopoeia (Part I, 2020 Version). The specific information of medicines is shown in Table 1. Eight commercial preparations of WDD were purchased from different medical corporations in China and Japan (Table 2).

**Table 1:** Detailed information on the medicines in the WDD

Official name	Botanical name	Part used	Production place (P.R. China)	Ancient dose (liang)	Modern dose (g)
Pinelliae rhizoma	*Pineilia ternata* (Thunb.) Breit	Tuber	Hebei	2	6
Bambusae caulis in taenias	*Phyllostachys nigra* (Lodd.) Munro var. *henonis* (Mitf.) Stapf ex Rendle	The middle layer of a stem	Guangdong, Sichuan, Anhui	2	6
Aurantii fructus immaturus	*Citrus aurantium* L	Young fruit	Jiangxi	2	6
Citri reticulatae pericarpium	*Citrus reticulata* Blanco	Mature pericarp	Hubei, Jiangxi, Shandong, Hebei	3	9
Glycyrrhizae radix et rhizoma	*Glycyrrhiza uralensis* Fisch	Root and rhizome	Neimenggu, Gansu, Ningxia, Xinjiang	1	3
Zingiberis rhizoma recens	*Zingbier officinale* Rosc	Steam and root	Sichuan	4	12

**Table 2:** Commercial preparations of WDD

No	Lot No	Purchase place	Sample type
CP1	CP9713050	Taoyuan, Taiwan, China	Concentrated powders
CP2	E085TU1	Taipei, Taiwan, China	Concentrated powders
CP3	A34227	Tainan, Taiwan, China	Concentrated particles
CP4	A210169220	New Territories, Hong Kong, China	Concentrated particles
CP5	21101235	New Taipei, Taiwan, China	Concentrated particles
CP6	HK200901	Kowloon, Hong Kong, China	Concentrated particles
CP7	YJ779	Osaka, Japan	Concentrated particles
CP8	EG657104	Kaohsiung, Taiwan, China	Concentrated particles

### Sample preparation

#### Preparation of the standard solution

Specific concentrations of naringin, hesperidin, neohesperidin, liquiritin, glycyrrhizic acid, adenosine, liquiritigenin, tangeretin, eriocitrin, naringenin and synephrine were dissolved in methanol to produce 11 reference standard store solutions.

#### Preparation of sample solutions

The pharmaceutical preparation technique of WDD material reference was performed according to ancient records. After preliminary research on the plant origin, producing area, harvesting period and dose conversion, the water for boiling, heating and filtration were also investigated. The following method was applied: Pinelliae Rhizoma (6.0 g), Bambusae Caulis in Taenias (6.0 g), Aurantii Fructus Immaturus (6.0 g), Citri Reticulatae Pericarpium (9.0 g), Glycyrrhizae Radix et Rhizoma (3.0 g) and Zingiberis Rhizoma Recens (12.0 g) were placed in a decoction pot and immersed in 1600 mL of deionized water for 30 min, followed by decocting once for 3 h first with high heat and then with low heat. The remaining decoction was filtered (100 mesh) to obtain a daily dose of WDD material reference. A total of 15 batches of WDD material references (No.: MR1-MR15) were prepared. It was determined that 25 mL of WDD extracted in the laboratory yielded approximately 2.625 g of medicine. Different weights of WDD commercial preparations (containing approximately 2.625 g of medicine) were dissolved in 25 mL of water. In total, 8 samples of commercial preparations were prepared.

#### Preparation of 6 herbal medicines in WDD

Each of the 6 herbal medications in WDD was extracted and prepared according to the same procedure as in the sample solution preparation process.

#### Preparation of quality control samples

The pooled quality control (QC) samples (No.: QC1-QC6) were obtained by mixing 50 μL of WDD material reference and eight commercial preparations to assess the reproducibility and stability of the developed method.

The sample solutions, standard solution, herbal medicines and QC samples were centrifuged at 14000 rpm for 10 min to obtain the supernatants. All samples were filtered through a 0.22 µm microporous membrane before determination.

### LC system

Ultra-performance liquid chromatography (UPLC) analysis was performed using a Waters ACQUITY UPLC™ system (Waters Corporation, Milford, MA, USA) provided with a dual solvent delivery system and an automatic sampler. Separation was performed on an Acquity UHPLC HSS T3 column (2.1 × 100 mm, 1.8 μm, Waters, Milford, USA) maintained at 35 °C, and the flow rate was 0.3 mL min^−1^ with a 2 μL injection volume. The mobile phases consisted of water (A) and acetonitrile (B) (both containing 0.1% formic acid, *v*/*v*), with the following linear elution gradient procedure: 0–5 min, 5–10% B; 5–17.5 min, 10–25% B; and 17.5–30 min, 25–80% B.

### Mass condition

A Waters SYNAPT G2 High Definition TOF mass spectrometer system (Waters, Manchester, UK) was linked to the UPLC system coupled with an electrospray ionization (ESI) source. Mass spectrometry was performed using the following operating conditions in both positive and negative ionization modes: mass range, *m/z* 50–1200 Da; capillary voltage, 2.5 kV; sample cone voltage, 40 V; ramp trap collision energy, 20–50 V; source temperature, 100 °C; desolvation temperature, 250 °C; desolvation gas (N_2_) flow, 800 L/h; and cone gas (N_2_) flow, 50 L/h. Quantification of 11 reference standards was accomplished using multiple reaction monitoring (MRM). The following study optimized the source sampling cone and collision energy for each constituent. The optimization results of the 11 compounds are shown in Additional file 1: Table S1. Leucine enkephalin (*m/z* 556.2771 in ESI^+^ and *m/z* 554.2615 in ESI^−^) was employed as an external reference for precise mass adjustment. The centroid mode data were gathered and analysed using Masslynx 4.1 software.

### Establishment of a chemical compound library of WDD

The specific data on chemical compounds separated from the 6 medicines in WDD were collected and analysed by searching databases such as the China National Knowledge Infrastructure (CNKI), PubMed, Web of Science, SciFinder and Traditional Chinese Medicine Systems Pharmacology Database and Analysis Platform (TCMSP). A self-establishing library of chemical components was set up by the UNIFI platform, including compound name, molecular formula, chemical structure (saved in “.mol” format), and accurate molecular mass.

### Data analysis by UNIFI software

The MS data were analysed by UNIFI software. A peak area larger than 200 was selected for 2D inspection. The parameters chosen for 3D peak detection were peak intensities of high energy over 150 counts and low energy over 1200 counts. A margin of error of 5 ppm was permitted for detecting compounds, and matching compounds would form anticipated pieces from the structure. Cross-adduct combinations were permitted for the positive adducts involving H^+^ and Na^+^ and negative adducts comprising HCOO^−^ and H^−^.

### Multivariate statistical analysis

Progenesis QI, an innovative data-processing platform, was used for complete data visualization and screening due to its high-throughput and high-sensitivity detection capabilities. Following data preparation, a temporary file including all biological information was automatically produced. After that, the enormous amounts of metabolic data were entered into the EZinfo 2.0 program for multivariate data analytics. The bioactive compound-guided cluster analysis method was established to evaluate the statistical relationship between the material reference and commercial preparations. The expression-based heatmap application from Heatmapper software was employed for hierarchical cluster analysis. The X-variables were composed of 23 batches of two forms of WDD samples in chromatographic fingerprints as columns, and the Y-variables were constructed with 11 bioactive markers as rows. In this research, the clustering method of centroid linkage and the distance measurement method of Pearson were used to evaluate the difference.

## Results and discussion

### UPLC‒MS characterization of chemical constituents from WDD

**Fig. 1:**
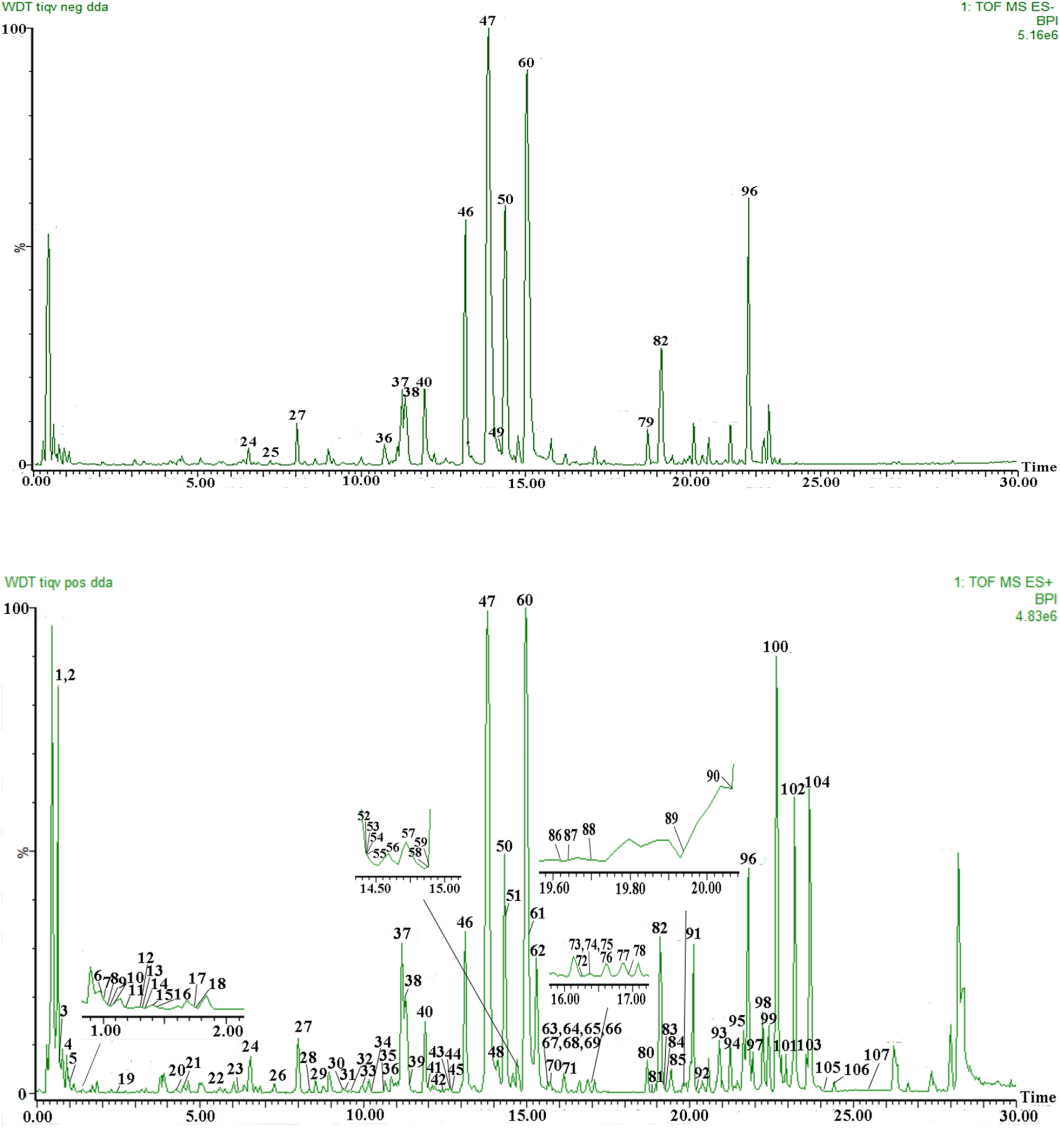
Bask peak ion chromatograms of WDD in negative and positive ESI modes

The high-resolution MS data of WDD were promptly collected using the UPLC-Q-TOF-MS^E^ technique. The base peak intensity (BPI) chromatograms of WDD in positive and negative ion modes are shown in Fig. 1. The BPI chromatograms of 6 single herbs of WDD are shown in Additional file 1: Figs. S1-S6.

### Fragmentation pattern study of WDD

The MS data were processed and analysed by UNIFI screening technology, and the fragment information was then automatically matched. Following further verification, a total of 107 compounds were confirmed in WDD, including 54 flavonoids, 14 triterpenoids, 10 organic acids, 7 alkaloids, 7 coumarins, and 15 other types. The comprehensive MS information of these compounds is summarized in Table 3. Furthermore, chemical structures were validated using precise mass, MS^E^ data, and relevant literature.

**Table 3:** Chromatographic and mass spectral data of the 107 compounds analysed by UPLC-QTOF-MS/MS

No	*t*_R_ (min)	Identification	Selected ion	Calculated mass (Da)	Measured mass (Da)	Mass error(ppm)	Formula	MS Fragmentation	Type	Source	Refs.
1^a^	0.63	Adenosine	[M + H]^+^	268.1033	268.1037	1.3	C_10_H_13_N_5_O_4_	136.0616[M + H-C_5_H_8_O_4_]^+^, 119.0345[M + H-C_5_H_8_O_4_-NH_3_]^+^	Alkaloid	PRP, AFI, BCT	[7]
2	0.69	Luteolin 3′,4′-dimethyl ether 7-*O*-rutinoside	[M + H]^+^	623.2001	623.1976	−4.0	C_29_H_34_O_15_	477.1390[M + H-Rha]^+^, 459.1351[M + H-Rha-H_2_O]^+^, 273.0624[M + H-Rha-Glc-CH_2_-CO]^+^	Flavonoid	AFI	[7]
3^a^	0.73	Synephrine	[M + H]^+^	168.1018	168.1018	−0.1	C_9_H_13_NO_2_	150.0901[M + H-H_2_O]^+^, 134.0593[M + H-H_2_O-CH_4_]^+^, 119.0499[M + H-H_2_O-NH_2_CH_3_]^+^, 107.0489[M + H-H_2_O-CHNHCH_3_]^+^	Alkaloid	CRP, AFI	[7]
4	0.97	Furfural	[M + H]^+^	97.0283	97.0283	−0.2	C_5_H_4_O_2_	81.0329[M + H–O]^+^, 69.0336[M + H–CO]^+^	Other type	PRP	[9]
5	1.00	Aminobenzoic acid	[M + H]^+^	138.0545	138.0544	−0.6	C_7_H_7_NO_2_	120.0439[M + H-H_2_O]^+^, 94.0651[M-COOH]^+^	Organic acid	PRP	[9]
6	1.01	Valine	[M + H]^+^	118.0858	118.0858	−0.4	C_5_H_11_NO_2_	102.0542[M + H-CH_4_]^+^, 100.0755[M + H-H_2_O]^+^, 88.0389[M + H-2CH_3_]^+^, 85.0283[M + H-CH_4_-NH_3_]^+^, 83.0482[M + H-NH_3_-H_2_O]^+^, 69.0336[M + H-NH_2_-CH_3_-H_2_O]^+^, 58.0659[M + H-CH_3_-HCOO]^+^	Organic acid	PRP	[9]
7	1.03	2-aminobutanoic acid	[M + Na]^+^	126.0540	126.0542	1.6	C_4_H_9_NO_2_	88.0389[M + H-CH_4_]^+^, 74.0243[M + H-C_2_H_6_]^+^, 69.0336[M + H-NH_3_-H_2_O]^+^, 58.0659[M + H-HCOOH]^+^	Organic acid	PRP	[9]
8	1.05	Hordenine	[M + H]^+^	166.1232	166.1237	3.0	C_10_H_15_NO	121.0338[M + H-NH(CH_3_)_2_]^+^	Other type	AFI	[7]
9	1.06	Pipecolic acid	[M + H]^+^	130.0861	130.0861	−0.2	C_6_H_11_NO_2_	112.0756[M + H-H_2_O]^+^, 94.0651[M + H-2H_2_O]^+^	Organic acid	PRP	[10]
10	1.08	6H-Purin-6-one,2-amino-1,9-dihydro-	[M + H]^+^	152.0564	152.0564	−0.3	C_5_H_5_N_5_O	174.0388[M + Na]^+^, 135.0290[M + H-NH_3_]^+^, 122.0235[M + H-NHCH_3_]^+^, 109.0504[M + H-NHCO]^+^	Alkaloid	PRP	[11]
11	1.19	Benzaldehyde	[M + H]^+^	107.0486	107.0486	−0.5	C_7_H_6_O	91.0540[M + H–O]^+^, 71.0385[M + H-HCOOH]^+^	Other type	PRP	[9]
12	1.29	Protocatechualdehyde	[M + H]^+^	139.0382	139.0381	−0.9	C_7_H_6_O_3_	121.0288[M + H-H_2_O]^+^, 109.0281[M + H-CH_2_O]^+^	Other type	PRP	[12]
13	1.31	Vanillic acid	[M + H]^+^	169.0492	169.0492	−0.3	C_8_H_8_O_4_	151.0383[M + H-H_2_O]^+^, 123.0431[M + H-HCOOH]^+^, 109.0286[M + H-CH_3_-COOH]^+^	Organic acid	CRP, AFI	[13, 14]
14	1.32	5-Hydroxymethyl furfual	[M + H]^+^	127.0388	127.0388	−0.2	C_6_H_6_O_3_	109.0281[M + H-H_2_O]^+^	Other type	AFI	[15]
15	1.40	*p*-Hydroxybenzaldehyde	[M + H]^+^	123.0433	123.0432	−0.9	C_7_H_6_O_2_	95.0487[M + H–CO]^+^	Other type	BCT	[7]
16	1.42	4-Acetylbenzoic acid	[M + H]^+^	165.0542	165.0542	−0.4	C_9_H_8_O_3_	147.0426[M + H-H_2_O]^+^	Organic acid	PRP	[16]
17	1.74	N-Methyltyramine	[M + H]^+^	152.1064	152.1063	−0.7	C_9_H_13_NO	103.0542[M + H-NH_2_CH_3_-H_2_O]^+^	Alkaloid	AFI	[7]
18	1.75	Phenylacetaldehyde	[M + H]^+^	121.0644	121.0643	−0.5	C_8_H_8_O	103.0542[M + H-H_2_O]^+^	Other type	PRP	[9]
19	2.43	Methyl 2-(methylamino) benzoate	[M + H]^+^	166.0866	166.0865	−0.9	C_9_H_11_NO_2_	137.0598[M + H-NHCH_2_]^+^, 136.0760[M + H-OCH_2_]^+^, 77.0389[M + H-NHCH_2_-COOH-CH_3_]^+^	Other type	CRP	[14]
20	4.37	Tryptophan	[M + H]^+^	205.0950	205.0944	−2.7	C_11_H_12_N_2_O_2_	188.0707[M + H-NH_3_]^+^, 143.0716[M + H-NH_2_-HCOOH]^+^, 142.0644[M + H-NH_3_-HCOOH]^+^, 118.0649[C_8_H_8_N]^+^	Alkaloid	GRR	[12]
21	4.67	Fabiatrin	[M + H]^+^	487.1542	487.1548	1.2	C_21_H_26_O_13_	341.0863[M + H-C_6_H_9_O_4_]^+^, 179.0347[C_9_H_7_O_4_]^+^	Coumarin	AFI	[17]
22	5.74	Luteolin 7-*O*-rutinoside	[M + H]^+^	595.1664	595.1664	0.2	C_27_H_30_O_15_	617.1539[M + Na]^+^, 577.1539[M + H-H_2_O]^+^, 287.0556[M + H-Rha-Glc]^+^, 153.0187[C_7_H_5_O_4_]^+^	Flavonoid	AFI	[7, 17]
23	6.24	3-Hydroxy-4-methoxybenzoic acid	[M + H]^+^	169.0488	169.0486	−0.9	C_8_H_8_O_4_	109.0280[M + H-CH_3_-COOH]^+^, 105.0329[M + H-H_2_O-HCOOH]^+^	Organic acid	CRP	[14]
24	6.60	Diosmetin 6,8-di-C-glucoside	[M-H]^−^	623.1612	623.1612	0.2	C_28_H_32_O_16_	533.1279[M-H-C_3_H_6_O_3_]^−^, 503.1146[M-H-C_4_H_8_O_4_]^−^, 443.1023[M-H-2C_3_H_6_O_3_]^−^, 413.0898[M-H-2C_3_H_6_O_3_-CO]^−^, 383.0763[M-H-2C_4_H_8_O_4_]^−^, 353.0666[M-H-2C_4_H_8_O_4_-CO]^−^	Flavonoid	CRP	[7]
25	7.14	Choerospondin	[M-H]^−^	433.1116	433.1121	1.2	C_21_H_22_O_10_	271.0603[M-H-Glc]^−^, 151.0024[C_8_H_7_O_3_]^−^	Flavonoid	GRR	[7]
26	7.19	Shaftoside	[M + H]^+^	565.1557	565.1563	1.1	C_26_H_28_O_14_	587.1317[M + Na]^+^, 547.1441[M + H-H_2_O]^+^	Flavonoid	GRR	[18]
27	8.12	Liquirtigenin	[M + H]^+^	257.0801	257.0799	−0.9	C_15_H_12_O_4_	239.0682[M + H-H_2_O]^+^, 137.0225[C_7_H_5_O_3_]^+^, 119.0487[M + H-C_7_H_6_O_3_]^+^	Flavonoid	GRR	[19–21]
28^a^	8.38	Quercetin	[M + H]^+^	303.0487	303.0482	−1.7	C_15_H_10_O_7_	285.0350[M + H-H_2_O]^+^, 257.0358[M + H-H_2_O-CO]^+^, 229.0487[M + H-C_2_H_2_O_3_]^+^, 181.0623[C_13_H_9_O]^+^, 153.0175[C_7_H_5_O_4_]^+^, 123.0444[C_7_H_7_O_2_]^+^, 109.0247[C_6_H_5_O_2_]^+^	Flavonoid	AFI, GRR	[19]
29	8.69	Naringenin-7-*O*-triglycoside	[M + H]^+^	743.2399	743.2394	−0.7	C_33_H_42_O_19_	595.1651[M + H-Rha]^−^	Flavonoid	AFI	[22]
30	9.39	Apiin	[M + H]^+^	565.1546	565.1539	−1.3	C_26_H_28_O_14_	433.1147[M + H-Api]^+^, 271.0613[M + H-Api-Glc]^+^	Flavonoid	AFI	[23]
31^a^	9.41	Eriocitrin	[M + H]^+^	597.1819	597.1825	1.0	C_27_H_32_O_15_	619.1599[M + Na]^+^, 579.1702[M + H-H_2_O]^+^, 451.1284[M + H-Rha]^+^, 435.1239[M + H-Rha-O]^+^, 433.1154[M + H-Rha-H_2_O]^+^, 289.0699[M + H-Rha-Glc]^+^, 163.0366[M + H-Rha-Glc-C_6_H_5_O_3_]^+^, 153.0176[M + H-Rha-Glc-C_8_H_7_O_2_]^+^	Flavonoid	AFI	[7, 24]
32	9.57	Naringenin-7-*O*-glucoside	[M + H]^+^	435.1290	435.1293	0.7	C_21_H_22_O_10_	273.0748[M + H-Glc]^+^	Flavonoid	AFI	[7, 22]
33	9.83	Eriodictyol	[M + H]^+^	289.0703	289.0702	−0.4	C_15_H_12_O_6_	179.0313[M + H-C_6_H_6_O_2_]^+^, 153.0174[M + H-C_8_H_8_O_2_]^+^, 135.0438[M + H-C_7_H_6_O_4_]^+^	Flavonoid	AFI, GRR	[7]
34	10.36	Orientin	[M + H]^+^	449.1070	449.1062	−1.7	C_21_H_20_O_11_	359.0851 [M + H-C_3_H_6_O_3_]^+^	Flavonoid	AFI	[7]
35	10.54	Isonaringin	[M + H]^+^	581.1866	581.1866	0.1	C_27_H_32_O_14_	603.1652[M + Na]^+^, 435.1255[M + H-Rha]^+^, 419.1331[M + H-Rha-O]^+^, 273.0754[M + H-Rha-Glc]^+^	Flavonoid	CRP, AFI	[23]
36	10.79	Ferulic acid	[M + H]^+^	195.0558	195.0554	−1.9	C_10_H_10_O_4_	177.0534[M + H-H_2_O]^+^, 134.0367[M + H-HCOOH-CH_3_]^+^, 117.0327[M + H-HCOOH-CH_2_-H_2_O]^+^	Organic acid	CRP, AFI	[13]
37^a^	11.18	Liquiritin	[M + H] +	419.1329	419.1323	−1.4	C_21_H_22_O_9_	257.0794[M + H-Glc]^+^, 239.0702[M + H-Glc-O]^+^	Flavonoid	GRR	[2, 3, 7, 21, 25]
38	11.32	Rutin	[M + H]^+^	611.1595	611.1577	−3.0	C_27_H_30_O_16_	303.0483[M + H-Rha-Glc]^+^, 285.0350[M + H-Rha-Glc-H_2_O]^+^	Flavonoid	AFI	[7, 22]
39	11.52	Pinocembrin	[M + H]^+^	257.0803	257.0802	−0.6	C_15_H_12_O_4_	239.0699[M + H-H_2_O]^+^, 221.0560[M + H-2H_2_O]^+^, 211.0740[M + H-H_2_O-CO]^+^	Flavonoid	GRR	[12]
40^a^	11.88	Naringenin	[M + H]^+^	273.0750	273.0747	−1.1	C_15_H_12_O_5_	255.0655[M + H-H_2_O]^+^, 179.0327[C_9_H_7_O_4_]^+^, 153.0178[M + H-C_8_H_8_O]^+^, 121.0509[C_4_H_9_O_4_]^+^, 85.0281[C_4_H_5_O_2_]^+^	Flavonoid	AFI, GRR	[7, 19]
41	11.95	Isosakuranetin-5,7-di-*O*-glucoside	[M + H]^+^	611.1976	611.1976	0.0	C_28_H_34_O_15_	449.1563[M + H-Glc]^+^, 287.0624[M + H-2Glc]^+^, 153.0191[C_19_H_27_O_14_-2Glc]^+^	Flavonoid	AFI, CRP	[7]
42	12.47	Kaempferol 3-*O*-rhamnoside	[M + H]^+^	433.1128	433.1128	−0.2	C_21_H_20_O_10_	415.0970[M + H-H_2_O]^+^, 397.0860[M + H-2H_2_O]^+^, 285.0375[M + H-Rha]^+^, 153.0176[C_7_H_5_O_4_]^+^	Flavonoid	AFI	[26]
43	12.72	Saponarin	[M + H]^+^	595.1651	595.1643	−1.4	C_27_H_30_O_15_	433.1088[M + H-Glc]^+^, 313.1809[M + H-Glc-C_4_H_8_O_4_]^+^	Flavonoid	GRR	[7]
44	12.73	Luteolin	[M + H]^+^	287.0540	287.0536	−1.4	C_15_H_10_O_6_	151.0384[C_8_H_7_O_3_]^+^, 107.0486[C_7_H_7_O]^+^	Flavonoid	AFI	[12]
45	12.75	Diosmetin-7-*O*-glucoside	[M + H]^+^	464.1222	463.1211	−2.4	C_22_H_22_O_11_	300.0615[M + H-Glc]^+^, 285.0722[M + H-Glc-O]^+^, 271.0577[M + H-Glc-CO]^+^	Flavonoid	AFI	[23]
46	13.18	Meranzin	[M + H]^+^	261.1112	261.1112	−0.1	C_15_H_16_O_4_	283.0926[M + Na]^+^, 243.0992[M + H-H_2_O]^+^, 189.0536[M + H-C_4_H_8_O]^+^, 131.0483[M + H-C_4_H_8_O-CO-CH_2_O]^+^, 103.0537[M + H-C_4_H_8_O-CH_2_O-2CO]^+^	Coumarin	AFI	[7, 27]
47^a^	13.81	Naringin	[M + H]^+^	581.1863	581.1863	−0.2	C_27_H_32_O_14_	603.1649[M + Na]^+^, 435.1256[M + H-Rha]^+^, 419.1319[M + H-Rha-O]^+^, 401.1165[M + H-Rha-O-H_2_O]^+^, 383.1111[M + H-Rha-O-2H_2_O]^+^, 273.0751[M + H-Rha-Glc]^+^, 153.0176[M + H-Rha-Glc-C_8_H_8_O]^+^, 121.0277[M + H-Rha-Glc-C_8_H_7_O_3_]^+^	Flavonoid	CRP, AFI	[4, 7, 17]
48	13.82	Nomilin	[M + H]^+^	515.2292	515.2281	−2.1	C_28_H_34_O_9_	469.6160[M + H-CH_2_O_2_]^+^, 411.3182[M + H-CH_2_O_2_-2CH_3_-H_2_O]^+^	Triterpenoid	AFI, CRP	[14, 17]
49	13.86	Nominin-17-*b*-D-glucoside	[M-H]^−^	693.2770	693.2758	−1.7	C_34_H_46_O_15_	529.2567[M-H-Glc]^−^	Triterpenoid	AFI	[22]
50^a^	14.31	Hesperidin	[M + H]^+^	611.1976	611.1976	0.0	C_28_H_34_O_15_	633.1789[M + Na]^+^, 465.1396[M + H-Rha]^+^, 449.1448[M + H-Rha-O]^+^, 431.1339[M + H-Rha-O-H_2_O]^+^, 303.0871[M + H-Rha-Glc]^+^	Flavonoid	CRP, AFI	[4, 7, 17, 28]
51	14.37	Meranzin hydrate	[M + H]^+^	279.1219	279.1216	−1.1	C_15_H_18_O_5_	261.1111[M + H-H_2_O]^+^, 189.0534[M + H-H_2_O-2CH_3_-CH_2_-CO]^+^	Coumarin	AFI	[13]
52	14.40	Apigenin-7-*O*-rutinoside	[M + H]^+^	579.1705	579.1700	−0.8	C_27_H_30_O_14_	417.1163[M + H-Rha-O]^+^, 271.0578[M + H-Rha-Glc]^+^	Flavonoid	AFI	[13]
53	14.41	3,4-Dihydroxybenzoic acid	[M + H]^+^	154.0218	154.0210	−5.0	C_7_H_6_O_4_	177.0174[M + Na]^+^, 111.0438[M + H-CO_2_]^+^, 93.0280[M + H-CO_2_-H_2_O]^+^	Organic acid	PRP, GRR, ZRR	[29]
54	14.42	Gallic acid	[M + H]^+^	171.0276	171.0273	−1.5	C_7_H_6_O_5_	153.0181[M + H-H_2_O]^+^, 135.0067[M + H-2H_2_O]^+^, 125.0227[M + H-HCOOH]^+^, 107.0123[M + H-HCOOH-H_2_O]^+^	Organic acid	PRP, AFI	[30]
55	14.43	Chrysin	[M + H]^+^	255.0643	255.0640	−1.2	C_15_H_10_O_4_	153.0181[C_7_H_5_O_4_]^+^	Flavonoid	ZRR	[26]
56	14.58	NomiliniCacid	[M + H]^+^	533.2387	533.2373	−2.6	C_28_H_36_O_10_	515.2302[C_28_H_35_O_9_]^+^, 473.2173[C_26_H_33_O_8_]^+^, 455.2074[C_26_H_31_O_7_]^+^, 429.2148[C_21_H_33_O_9_]^+^, 393.2117[C_18_H_33_O_9_]^+^, 379.2070[C_23_H_29_O_4_]^+^	Triterpenoid	AFI	[17]
57	14.66	Diosmin	[M + H]^+^	609.1816	609.1818	0.4	C_28_H_32_O_15_	463.1166[M + H-Rha]^+^, 301.0692[M + H-Rha-Glc]^+^, 268.0460[M + H-Rha-Glc-CH_3_]^+^	Flavonoid	AFI	[7]
58^a^	14.94	Hesperetin	[M + H]^+^	303.0861	303.0859	−0.5	C_16_H_14_O_6_	327.0845[M + Na]^+^, 153.0178[C_7_H_5_O_4_]^+^, 149.0604[C_9_H_11_O_2_-2H]^+^, 117.0331[C_9_H_11_O_2_-2H-CH_2_-H_2_O]^+^,	Flavonoid	AFI, GRR	[7, 17, 19, 24]
59	14.94	Didymin	[M + H]^+^	595.1953	595.1961	1.3	C_28_H_34_O_14_	617.1854[M + Na]^+^, 559.1815[M + H-2H_2_O]^+^, 447.1259[M + H-Rha]^+^, 431.1330[M + H-Rha-O]^+^, 413.1200[M + H-H_2_O-Rha-O]^+^, 285.0738[M + H-Rha-Glc]^+^	Flavonoid	AFI	[13]
60^a^	14.98	Neohesperidin	[M + H]^+^	611.1815	611.1819	0.7	C_28_H_34_O_15_	633.1784[M + Na]^+^, 593.1866[M + H-H_2_O]^+^, 465.1396[M + H-Rha]^+^, 449.1448[M + H-Rha-O]^+^, 431.1339[M + H-Rha-O-H_2_O]^+^, 303.0868[M + H-Rha-Glc]^+^, 285.0761[M + H-Rha-Glc-H_2_O]^+^	Flavonoid	CRP, AFI	[4, 7, 17]
61	14.99	Byakangelicol	[M + H]^+^	317.1025	317.1023	−0.6	C_17_H_16_O_6_	273.0771[C_15_H_13_O_5_]^+^, 233.0115[C_11_H_5_O_6_]^+^, 231.0403[C_16_H_7_O_2_]^+^	Coumarin	GRR	[19]
62	15.30	Isorhamnetin	[M + H]^+^	317.0644	317.0639	−1.6	C_16_H_12_O_7_	302.0407[M + H-CH_3_]^+^	Flavonoid	AFI	[13]
63	15.62	Esculetin	[M + H]^+^	179.0333	179.0332	−0.6	C_9_H_6_O_4_	151.0384[M + H–CO]^+^, 133.0280[M + H-HCOOH]^+^, 123.0437[M + H-C_2_O_2_]^+^, 105.0329[M + H-C_2_O_2_-H_2_O]^+^	Coumarin	AFI	[17, 26]
64	15.62	Umbelliferone	[M + H]^+^	163.0376	163.0373	−1.7	C_9_H_6_O_3_	135.0433[M + H–CO]^+^, 119.0484[M + H-CO_2_]^+^	Coumarin	AFI	[7]
65	15.62	Kaempferol	[M + H]^+^	287.0545	287.0543	−0.7	C_15_H_10_O_6_	241.0471[M + H-CH_2_O_2_]^+^, 91.0540[M + H-C_8_H_4_O_6_]^+^	Flavonoid	AFI	[26]
66	15.62	Trifolirhizin	[M + H]^+^	447.1281	447.1277	−0.9	C_22_H_22_O_10_	429.1167[M + H-H_2_O]^+^, 411.1043[M + H-2H_2_O]^+^, 393.0968[M + H-3H_2_O]^+^, 285.0751[M + H-Glc]^+^	Flavonoid	GRR	[12]
67	15.62	Diethyl oxalate	[M + H]^+^	147.0653	147.0653	0.1	C_6_H_10_O_4_	129.0542[M + H-H_2_O]^+^, 83.0126[M + H-H_2_O-C_2_H_5_O]^+^, 68.9972[M + H-H_2_O-C_2_H_5_O-CH_3_]^+^	Other type	PRP	[9]
68	15.62	Phthalic anhydride	[M + H]^+^	149.0228	149.0227	−0.6	C_8_H_4_O_3_	133.0280[M + H–O]^+^, 121.0279[M + H–CO]^+^, 105.0329[M-COOH]^+^	Other type	PRP	[11, 16]
69	15.62	2-Methoxy-4-vinylphenol	[M + H]^+^	151.0747	151.0746	−0.6	C_9_H_10_O_2_	136.0490[M + H-CH_3_]^+^, 123.0437[M + H-C_2_H_4_]^+^, 119.0484[M + H-CH_3_-O]^+^, 117.0332[M + H-H_2_O-CH_4_]^+^	Other type	CRP	[14]
70	15.65	Luteone	[M + H]^+^	355.1564	355.1557	−1.9	C_20_H_18_O_6_	311.0527[M + H-C_3_H_8_]^+^, 221.0424[C_11_H_9_O_5_]^+^	Flavonoid	GRR	[7]
71	16.11	Isosakuranin	[M + H]^+^	449.1461	449.1448	−2.9	C_22_H_24_O_10_	419.1358[M + H-CH_2_O]^+^, 287.0924[M + H-Glc]^+^, 257.0879[M + H-Glc-CH_2_O]^+^, 153.0178[C_14_H_20_O_8_-Glc]^+^	Flavonoid	AFI	[7]
72	16.25	Neoliquiritin	[M + H]^+^	419.1329	419.1323	−1.4	C_21_H_22_O_9_	257.0794[M + H-Glc]^+^, 239.0702[M + H-Glc-O]^+^	Flavonoid	GRR	[7]
73	16.38	Obacunone	[M + H]^+^	455.2057	455.2051	−1.4	C_26_H_30_O_7_	437.1988[M + H-H_2_O]^+^, 411.2189[M + H-CO_2_]^+^, 409.2007[M + H–CO-H_2_O]^+^, 393.2066[M + H-CO_2_-H_2_O]^+^, 391.1943[M + H–CO-2H_2_O]^+^, 349.1822[M + H–CO-2H_2_O-C_3_H_6_]^+^	Triterpenoid	AFI	[7, 17]
74	16.38	Deacetyl nomilin	[M + H]^+^	473.2159	473.2150	−2.0	C_26_H_32_O_8_	455.2053[M + H-H_2_O]^+^	Triterpenoid	AFI	[7]
75	16.38	Isosarotanoside	[M-H]^−^	563.1747	563.1765	3.2	C_27_H_32_O_13_	609.1808[M + COOH]^−^, 255.0137[M + H-Rha-Glc]^−^	Flavonoid	CRP	[7]
76	16.71	Isoliquiritin apioside	[M + H]^+^	551.1784	551.1765	−3.4	C_26_H_30_O_13_	257.8640[M + H-Api-Glc]^+^, 147.2897[C_7_H_15_O_3_]^+^, 137.0648[C_8_H_9_O_2_]^+^	Flavonoid	PRP, GRR	[7]
77	16.78	Licochalcone B	[M + H]^+^	287.0919	287.0921	0.7	C_16_H_14_O_5_	271.0863[C_12_H_15_O_7_]^+^	Flavonoid	GRR	[31]
78	16.79	Ononin	[M + H]^+^	431.1327	431. 1319	−1.8	C_22_H_22_O_9_	476.2991[M-H + HCOOH]^+^, 269.0797 [M + H-Glc]^+^	Flavonoid	GRR	[7, 20]
79	18.50	Uralsaponin N	[M-H]^−^	837.3916	837.3909	−0.8	C_42_H_62_O_17_	661.3422[M-H-GluA]^−^, 485.3456[M-H-2GluA]^−^, 467.2256[M-H-2GluA-H_2_O]^−^, 449.3533[M-H-2GluA-2H_2_O]^−^	Triterpenoid	GRR	[7]
80	18.56	Calycosin	[M + H]^+^	285.0746	285.0741	−1.7	C_16_H_12_O_5_	213.0538[M + H-2CO-CH_4_]^+^	Flavonoid	GRR	[7]
81	18.85	CitrusinIII	[M + H]^+^	728.3994	728.3983	−1.5	C_36_H_53_N_7_O_9_	700.3988[M + H–CO]^+^	Alkaloid	CRP	[7]
82	19.10	Liquiritin apioside	[M + H]^+^	551.1742	551.1721	−3.8	C_26_H_30_O_13_	389.1217[M + H-Glc]^+^, 257.0789[M + H-Glc-Api]^+^	Flavonoid	GRR, PRP	[7, 20]
83	19.14	Uralsaponin F	[M + H]^+^	897.4120	897.4120	−0.1	C_44_H_64_O_19_	545.3578[M + H-2GluA]^+^, 527.3006[M + H-2GluA-H_2_O]^+^, 497.3338[M + H-2GluA-H_2_O-CH_2_O]^+^	Triterpenoid	GRR	[7]
84	19.27	Glycycoumarin	[M + H]^+^	369.1325	369.1320	−1.3	C_21_H_20_O_6_	353.1026[M + H-CH_4_]^+^, 315.0827[M + H-C_4_H_6_]^+^, 299.0888[M + H-C_4_H_6_-O]^+^, 285.0753[M + H-C_4_H_6_-CH_2_O]^+^	Coumarin	GRR	[13]
85	19.36	Glycyrrhetinic acid	[M + H]^+^	855.4024	855.4014	−1.2	C_42_H_62_O_18_	679.3223[M + H-GluA]^+^, 503.3375[M + H-2GluA]^+^, 467.3128[M + H-2GluA-2H_2_O]^+^	Triterpenoid	GRR	[20]
86	19.62	Lupiwighteone	[M + H]^+^	339.1218	339.1214	−1.3	C_20_H_18_O_5_	283.0579[M + H-C_4_H_8_]^+^, 255.0645[M + H-C_4_H_8_-CO]^+^, 165.0170[C_12_H_13_O_4_-C_4_H_8_]^+^, 135.0436[C_8_H_7_O_2_]^+^	Flavonoid	GRR	[7]
87	19.64	Melitidin	[M + H]^+^	725.2293	725.2293	−0.1	C_33_H_40_O_18_	419.1338[C_21_H_23_O_9_]^+^, 404.1101[C_21_H_23_O_9_-CH_3_]^+^, 389.0856[C_21_H_23_O_9_-2CH_3_]^+^	Flavonoid	AFI, CRP	[7]
88	19.70	Poncirin	[M + H]^+^	595.2019	595.2015	−0.6	C_28_H_34_O_14_	617.1823[M + Na]^+^, 449.1407[M + H-Rha]^+^, 433.1495[M + H-Rha-O]^+^, 397.1251[M + H-Rha-O-2H_2_O]^+^, 287.0908[M + H-Rha-Glc]^+^	Flavonoid	AFI	[4, 7]
89	19.94	Licorice saponin A3	[M + H]^+^	985.4567	985.4550	−1.7	C_48_H_72_O_21_	809.42744[M + H-GluA]^+^, 647.3741[M + H-GluA-Glc]^+^, 633.3950[M + H-2GluA]^+^, 615.3851[M + H-2GluA-H_2_O]^+^, 471.3466[M + H-2GluA-Glc]^+^, 453.3351[M + H-2GluA-Glc-H_2_O]^+^, 435.3224[M + H-2GluA-Glc-2H_2_O]^+^, 407.3284[M + H-2GluA-Glc-2H_2_O-CO]^+^	Triterpenoid	GRR	[9]
90	20.06	Riparin Ι	[M + H]^+^	256.1349	256.1338	−4.3	C_16_H_17_NO_2_	121.1011[C_8_H_9_O]^+^, 105.0213[C_7_H_5_O]^+^	Alkaloid	AFI	[7]
91	20.10	22β-Acetoxyl-glycyrrhizin	[M + H]^+^	881.4181	881.4171	−1.1	C_44_H_64_O_18_	529.3373[M + H-2GluA]^+^, 511.3416[M + H-2GluA-H_2_O]^+^, 451.3151[M + H-2GluA-H_2_O-CH_3_COOH]^+^, 433.3008[M + H-2GluA-2H_2_O-CH_3_COOH]^+^	Triterpenoid	GRR	[7]
92	20.23	Isosakuranetin	[M + H]^+^	287.0912	287.0912	0.2	C_16_H_14_O_5_	153.0186[C_7_H_5_O_4_]^+^, 133.0795[C_9_H_9_O]^+^	Flavonoid	CRP	[14]
93	21.15	5,7,8,4'-Tetramethoxyflavone	[M + H]^+^	343.1182	343.1176	−1.7	C_19_H_18_O_6_	313.0709[M + H-2CH_2_]^+^	Flavonoid	AFI	[22]
94	21.54	4′-*O*-methyl glabridin	[M + H]^+^	355.1513	355.1498	−4.2	C_21_H_22_O_5_	287.0994[M + H-C_5_H_8_]^+^, 283.0584[M + H-C_4_H_9_-CH_3_]^+^, 272.0595[M + H-C_5_H_8_-CH_3_]^+^	Flavonoid	GRR	[12]
95	21.55	M-Cymene	[M + H]^+^	135.1160	135.1159	−0.9	C_10_H_14_	91.0540[M + H-C_3_H_8_]^+^, 77.0386[M + H-C_3_H_8_-CH_3_]^+^	Other type	ZRR	[26]
96^a^	21.79	Glycyrrhizic acid	[M + H]^+^	823.4116	823.4116	0.0	C_42_H_62_O_16_	845.3920[M + Na]^+^, 647.3798[M + H-GluA]^+^, 471.3476[M + H-2GluA]^+^, 453.3371[M + H-2GluA-H_2_O]^+^, 435.3252[M + H-2GluA-2H_2_O]^+^, 407.3302[M + H-2GluA-2H_2_O-CO]^+^	Triterpenoid	GRR	[4, 17]
97	21.82	Licorice saponin G2	[M + H]^+^	839.4058	839.4048	−1.2	C_42_H_62_O_17_	663.3780[M + H-GluA]^+^, 487.3412[M + H-2GluA]^+^, 469.3293[M + H-2GluA-H_2_O]^+^, 451.3190[M + H-2GluA-2H_2_O]^+^, 439.3181[M + H-2GluA-CH_2_O]^+^	Triterpenoid	GRR	[7, 21]
98	22.20	Nobiletin	[M + H]^+^	403.1386	403.1385	−0.3	C_21_H_22_O_8_	425.1197[M + Na]^+^, 387.1055[M + H-CH_4_]^+^, 373.0916[M + H-2CH_3_]^+^, 358.0673[M + H-3CH_3_]^+^, 345.0952[M + H-2CH_3_-CO]^+^, 327.0851[M + H-2CH_3_-H_2_O-CO]^+^, 321.0698[M + H-4CH_2_-CO_2_]^+^, 211.0225[C_11_H_13_O_6_-2CH_3_]^+^, 183.0289[C_11_H_13_O_6_-2CH_3_-CO]^+^, 163.0744[C_10_H_11_O_2_]^+^	Flavonoid	CRP	[7, 17]
99	22.38	Uralsaponin C	[M + H]^+^	824.4180	824.4139	−5.0	C_42_H_64_O_16_	663.3671[M + H-GluA]^+^, 473.3512[M + H-2GluA]^+^, 457.3387[M + H-2GluA-O]^+^, 455.3419[M + H-2GluA-H_2_O]^+^	Triterpenoid	GRR	[12]
100	22.61	16-Dihydrosphingosine	[M + H]^+^	274.2746	274.2742	−1.5	C_16_H_35_NO_2_	256.2610[M-H_2_O]^+^	Other type	PRP	[16]
101	22.81	Limonin	[M + H]^+^	471.2004	471.1995	−1.9	C_26_H_30_O_8_	493.1798[M + Na]^+^, 453.1877[M + H-H_2_O]^+^, 435.1806[M + H-2H_2_O]^+^, 425.1955[M + H–CO-H_2_O]^+^	Triterpenoid	AFI	[7, 17]
102	23.20	Isosinensetin	[M + H]^+^	373.1278	373.1275	−0.7	C_20_H_20_O_7_	357.1425[M + H-CH_4_]^+^, 343.0747[M + H-2CH_3_]^+^, 327.0475[M + H-2CH_3_-CH_4_]^+^, 315.0854[M + H-2CH_3_-CO]^+^, 163.0746[C_10_H_11_O_2_]^+^	Flavonoid	AFI	[7]
103^a^	23.55	6-Gingerol	[M + H]^+^	295.0247	295.0240	−2.4	C_17_H_26_O_4_	317.1713[M + Na]^+^, 277.1777[M + H-H_2_O]^+^, 259.1012[M + H-2H_2_O]^+^, 179.0635[C_10_H_11_O_3_]^+^, 177.1246[C_11_H_13_O_2_]^+^, 137.0593[C_8_H_9_O_2_]^+^	Other type	ZRR	[13]
104	23.83	3,5,6,7,8,3′,4′-Heptamethoxyflavone	[M + H]^+^	433.1491	433.1489	−0.4	C_22_H_24_O_9_	455.1309[M + Na]^+^, 417.1170[M + H-CH_4_]^+^, 403.1018[M + H-2CH_3_]^+^, 388.0773[M + H-3CH_3_]^+^, 385.0914[M + H-2CH_3_-H_2_O]^+^, 373.0511[M + H-2CH_3_-CO]^+^, 360.0847[M + H-3CH_3_-CO]^+^	Flavonoid	CRP, AFI	[7, 22, 32]
105	24.18	5-Hydroxy-3,6,7,8,3',4'-Hexamethoxyflavone	[M + H]^+^	419.1329	419.1323	−1.4	C_21_H_22_O_9_	441.1142[M + Na]^+^, 389.0858[M + H-CH_3_]^+^, 371.0728[M + H-2CH_3_-H_2_O]^+^, 361.0907[M + H-2CH_3_-CO]^+^, 346.0662[M + H-3CH_3_-CO]^+^, 328.0563[M + H-3CH_3_-CO-H_2_O]^+^	Flavonoid	AFI	[23]
106^a^	24.28	Tangeretin	[M + H]^+^	373.1276	373.1273	−0.9	C_20_H_20_O_7_	395.1074[M + Na]^+^, 357.0945[M + H-CH_4_]^+^, 343.0806[M + H-2CH_3_]^+^, 328.0563[M + H-3CH_3_]^+^, 315.0847[M + H-2CH_3_-CO]^+^, 300.0619[M + H-3CH_3_-CO]^+^, 299.0536[M + H-4CH_2_-H_2_O]^+^, 297.0748[M + H-2CH_3_-CH_4_-H_2_O]^+^, 211.0318[C_11_H_13_O_6_-2CH_3_]^+^, 183.0288[C_11_H_13_O_6_-2CH_3_-CO]^+^	Flavonoid	CRP	[7, 17]
107	25.37	Ethylparaben	[M + H]^+^	167.0695	167.0694	−0.8	C_9_H_10_O_3_	149.0595[M + H-H_2_O]^+^, 121.0271[M-CH_2_CH_3_-H_2_O]^+^, 95.0487[M-COOC_2_H_5_]^+^, 77.0384[M-COOC_2_H_5_-H_2_O]^+^	Other type	PRP	[9]

*PRP* Pinelliae Rhizoma Praeparatum, *AFI* Aurantii Fructus Immaturus, *CRP* Citri Reticulatae Pericarpium, *BCT* Bambusae Caulis in Taenias, *GRR* Glycyrrhizae Radix et Rhizoma, *ZRR* Zingiberis Rhizoma Recens

^a^Identified by comparison with reference standards

### Identification of flavonoids

Flavonoids and their glycosides are abundant in WDD and are widely present in plant material. In this study, the matching of mass spectral data with the UNIFI analytical platform authenticated a total of 54 flavonoids, including 16 flavones, 7 flavonols, 26 flavanones, 1 isoflavanone, 3 chalcones and 1 flavanol.

The glycosidic linkages joined by oxygen atoms in flavonoid glycosides may be cleaved in both positive and negative ion modes, with the majority of them characterized by neutral losses, for example, 162 Da (Glc), 146 Da (Rha) and 132 Da (Api) [33]. The cross-ring cleavages of flavone C-glycosides of saccharidic residues produced the primary product ions. Therefore, it was simple to lose C_2_H_4_O_2_ (60 Da), C_3_H_6_O_3_ (90 Da), and C_4_H_8_O_4_ (120 Da) groups from the precursor ions [7]. Compound 60 revealed a quasimolecular [M + H]^+^ ion at *m/z* 611.1879 (C_28_H_34_O_15_). The fragment ions at *m/z* 465.1396 ([M + H-Rha]^+^), 449.1448 ([M + H-Rha-O]^+^), 431.1339 ([M + H-Rha-O-H_2_O]^+^), 303.0868 ([M + H-Rha-Glc]^+^), 285.0761 ([M + H-Rha-Glc-H_2_O]^+^) were produced by removing a molecule of rhamnose and a molecule of glucose, respectively. Therefore, it was recognized as neohesperidin by analysing the reference substance, and its fragmentation behaviour is shown in Additional file 1: Fig. S7. Compound 24 exhibited a parent ion [M-H]^−^ at *m/z* 623.1612, which generated fragmentation ions at *m/z* 533.1279 ([M-H-C_3_H_6_O_3_]^−^), 503.1146 ([M-H-C_4_H_8_O_4_]^−^), 443.1023 ([M-H-2C_3_H_6_O_3_]^−^), 413.0898 ([M-H-C_4_H_8_O_4_-C_3_H_6_O_3_]^−^), and 383.0763 ([M-H-C_4_H_8_O_4_-C_3_H_6_O_3_-CO]^−^) by the elimination of the CO moiety. These results indicated that the fragmentation patterns were comparable to those of diosmetin 6,8-di-C-glucoside. The mass spectrum and possible fragmentation pathways of diosmetin 6,8-di-C-glucoside in negative ion mode are shown in Additional file 1: Fig. S8.

It is widely known that the RDA fragmentation processes as well as losses of small molecules and radicals, including CH_3_, CO, and CO_2_, are the primary MS behaviours of flavone aglycones. Furthermore, neutral loss of CH_4_ (16 Da) was produced in the presence of an ortho-methoxyl substituent group. Compound 104 displayed a [M + H]^+^ ion at *m/z* 433.1489 (C_22_H_24_O_9_) with diagnostic ions at *m/z* 417.1170 and 403.1018 via the loss of 16.0313 Da (CH_4_) and 30.0470 Da (2CH_3_), respectively. Moreover, fragment ions at *m/z* 388.0773 ([M + H-3CH_3_]^+^), 385.0914 ([M + H-2CH_3_-H_2_O]^+^), 373.0511 ([M + H-2CH_3_-CO]^+^), and 360.0847 ([M + H-3CH_3_-CO]^+^) were also observed. Thus, this compound was identified as 3,5,6,7,8,3′,4′-heptamethoxyflavone, and fragmentation patterns are shown in Additional file 1: Fig. S9.

### Identification of triterpenoids

A total of 14 triterpenoids were identified in WDD, including 8 pentacyclic triterpenoids from GRR and 6 limonoids from AFI and CRP.

Triterpenoid saponins in GRR were made up of oleanane-type triterpene sapogenins and saccharide groups via the hydroxyl group at the C-3 position, with glucose and glucuronic acid being the most frequent saccharides. As a result, the saccharide groups in the structures were identified using the neutral losses of the sugar moiety. Compound 96 was positively identified as glycyrrhizic acid using a reference standard. The MS fragmentation pattern of glycyrrhizic acid was studied in depth to contribute to the characterization of these pentacyclic triterpenoids (Additional file 1: Fig. S10). Glycyrrhizic acid gave an [M + H]^+^ ion at *m/z* 823.4116, along with five major fragment ions at *m/z* 647.3798 ([M + H-GluA]^+^), 471.3476 ([M + H-2GluA]^+^), 453.3371 ([M + H-2GluA-H_2_O]^+^), 435.3252 ([M + H-2GluA-2H_2_O]^+^), and 407.3302 ([M + H-2GluA-2H_2_O-CO]^+^) observed in the high-energy MS^E^ spectra.

Limonoids were created by removing four terminal carbons from the side chain of an apotirucallane or apoeuphane skeleton and then cyclizing them to generate the 17-furan ring [7]. Additionally, some compounds lost complicated groups, such as CO, CO_2_, H_2_, and others. Compound 73 gave a hydrogenated molecule [M + H]^+^ at *m/z* 455.2051 and produced predominant fragment ions at m/z 437.1988 ([M + H-H_2_O]^+^), 411.2189 ([M + H-CO_2_]^+^), 409.2007 ([M + H–CO-H_2_O]^+^), 393.2066 ([M + H-CO_2_- H_2_O]^+^), 391.1943 ([M + H–CO-2H_2_O]^+^), and 349.1822 ([M + H–CO-2H_2_O- C_3_H_6_]^+^) in positive ion mode. It was identified as obacunone (Additional file 1: Fig. S11).

### Identification of organic acids

A total of 10 organic acids were found in positive ion mode and originated from five of six Chinese medicines, except BCT. Organic acids, which are mainly extracted with polar solvents, are the abundant chemical constituents of Pinellia rhizoma species [34]. [M-CH_3_]^+^, [M-H_2_O]^+^ and [M-HCOOH]^+^ in the mass spectra of organic acids showed the presence of a polyhydroxy molecule comprising carboxylic acid groups. Compound 53 produced an [M + H]^+^ ion at *m/z* 154.0210 with the chemical Formula C_7_H_6_O_4_. The main ions emerged at 111.0438 ([M + H-CO_2_]^+^) and 93.0710 ([M + H-CO_2_-H_2_O]^+^), which corresponds to the usual structure of organic acids. Thus, Compound 53 was considered to be 3,4-dihydroxybenzoic acid (Additional file 1: Fig. S12).

### Identification of alkaloids

Seven alkaloids in WDD were derived from five of six Chinese medicines, except ZRR. According to the literature, the principal fragment patterns of alkaloids are neutral losses, such as methyl radicals, hydrogen radicals, and CO, caused by the serial cleavage of substituted methoxyl or methylenedioxyl groups on the A- and D-rings [35]. Component 3 exhibited a quasimolecular ion at *m/z* 168.1018 (C_9_H_13_NO_2_). The fragment of synephrine was suggested by the diagnostic ions at *m/z* 150.0901 ([M + H-H_2_O]^+^), 134.0593 ([M + H-H_2_O-CH_4_]^+^), 119.0499 ([M + H-H_2_O- NH_2_CH_3_]^+^), and 107.0489 ([M + H-H_2_O-CHNHCH_3_]^+^) in the spectrum (Additional file 1: Fig. S13).

### Identification of coumarins

Seven coumarins were identified in WDD, mostly from AFI and GRR. The basic fragmentation mechanism of coumarins involves the losses of OH, CH_3_, CO and CO_2_ [36]. The precursor ion [M + H]^+^ at *m/z* 261.1112 (C_15_H_16_O_4_), which was recognized as Compound 46, was confirmed to be meranzin by the fragment ions at *m/z* 243.0992 ([M + H-H_2_O]^+^), 189.0536 ([M + H-C_4_H_8_O]^+^), 131.0483 ([M + H-C_4_H_8_O-CO-CH_2_O]^+^), 103.0537 ([M + H-C_4_H_8_O-CH_2_O-2CO]^+^) (Additional file 1: Fig. S14).

### Identification of other types

This group includes certain chemicals with fewer species and lower concentrations. The mass spectra data detected from the MassLynx workstation were compared with UNIFI software, and the results were confirmed by a literature study. A total of 15 compounds were deduced. Component 103 displayed a molecular ion [M + H]^+^ at *m/z* 295.0240 (C_17_H_26_O_4_). The characteristic ions at *m/z* 277.1777 ([M + H-H_2_O]^+^), 259.1012 ([M + H-2H_2_O]^+^), 179.0635 ([C_10_H_11_O_3_]^+^), 177.1246 ([C_11_H_13_O_2_]^+^), 137.0593 ([C_8_H_9_O_2_]^+^) were generated. Therefore, the component was unequivocally identified as 6-gingerol with the reference material (Additional file 1: Fig. S15).

### Multivariate statistical analysis

In terms of QTOF-MS, it was discovered that positive ion mode may provide more sensitive and accurate mass spectra in terms of UPLC-QTOF-MS. Furthermore, the positive ion mode made it simpler to validate molecular ions in the identification of each signal. As a result, the UPLC-QTOF-MS data for the multivariate statistical analysis were collected in positive ion mode.

Few technical and analytical mistakes in UPLC-QTOF-MS-based metabolomics can prevent interference with multivariate statistical analysis to generate trustworthy and high-quality results. The QC sample was also evaluated in tandem with the WDD samples to ensure system stability. The data quality was assessed by comparing all QC samples.

**Fig. 2:**
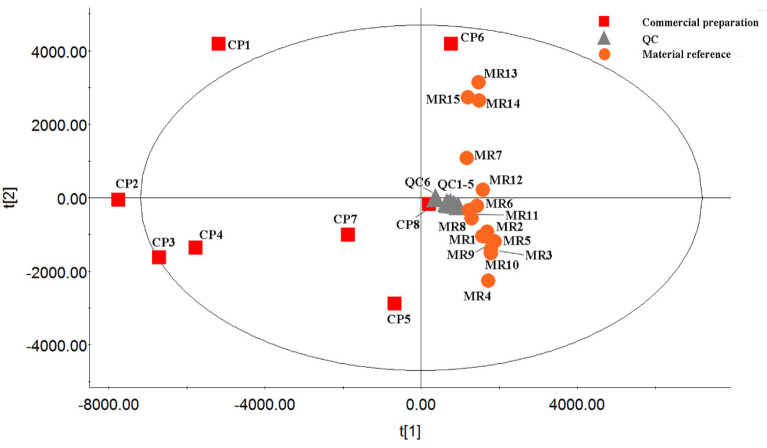
PCA score plot of material reference and commercial preparation

Samples of the WDD material reference and its commercial formulations were imported into progenesis QI for principal component analysis (PCA). The scatterplot of PCA is shown in Fig. 2. QC samples are spread around the origin and are closely aggregated. The findings suggest that the substantial differences identified by multivariate statistical analysis between material reference and commercial preparation were more likely to be the consequence of composition changes rather than artefacts resulting from technical faults. R^2^ (cum) is a popular metric for evaluating the quality of a PCA model, with values near 1.0 indicating strong fitness and predictive performance. In this study, R^2^X (cum) is 0.9, showing that the developed PCA model has acceptable fitness and prediction. The 23 samples were almost evenly split into two groups, indicating a difference in quality between the WDD material reference and its commercial preparations.

Modern pharmacological studies have shown that WDD mainly has lipid-lowering, anti-inflammatory, anti-schizophrenia, protection against cell damage and other effects. Eleven bioactive ingredients with major pharmacological effects were screened from WDD. Liquiritigenin, liquiritin, glycyrrhizic acid, naringin and tangeretin have anti-inflammatory effects by decreasing the synthesis of IL-6, TNF-α and VEGF [37–42]. Eriocitrin and liquiritin regulate the expression of Nrf2 and NF-κB, thereby downregulating inflammation and oxidative stress [38, 43]. Synephrine, liquiritigenin, hesperidin, neohesperidin and tangeretin possess lipid-lowering effects by regulating the insulin receptor (IR) and suppressing adipocyte differentiation [44–48]. Liquiritigenin has the ability to cure immunological dysfunction by increasing cAMP synthesis in several cell lines and controlling immune cell death [40, 49]. Adenosine alleviated amyloid β-protein_25-35_-induced brain damage by preventing apoptosis and oxidative stress [50]. Naringenin may have anti-coronavirus disease 2019 (COVID-19) effects by inhibiting the COVID-19 major protease 3-chymotrypsin-like protease (3CLpro) and decreasing angiotensin converting enzyme receptor activation [51].

**Fig. 3:**
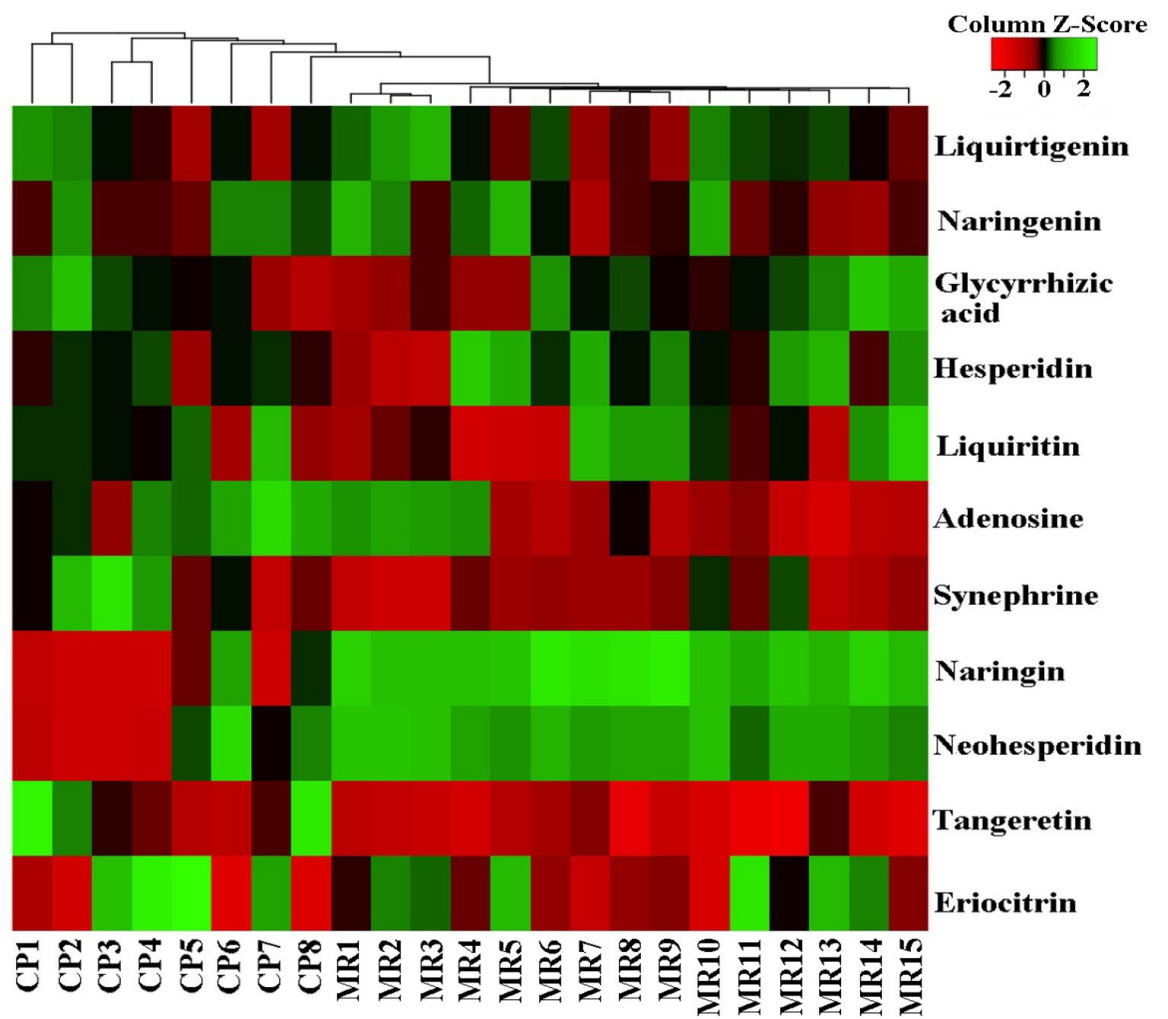
Heatmap of the 11 chemical constituents of WDD prepared in the laboratory and its 8 commercial preparations. MR1-MR15 were 15 batches of WDD material reference. CP1-CP8 are commercial preparations of WDD

The contents of 11 components in the WDD material reference and its commercial prescriptions were normalized by a heatmap (Fig. 3). Clearly, WDD samples in the material reference gathered into one cluster according to the hierarchical cluster analysis. There was no clear aggregation trend among the commercial WDD, but there was an obvious correlation with the material reference, that is, CP8. Overall, among the 11 components analysed, the contents of neohesperidin and naringin in the WDD material reference were higher than those in its commercial formulas, adenosine and synephrine in the WDD material reference were lower than those in its commercial formulas, while the contents of the other components were generally consistent. Generally, the differences between the WDD material reference and its commercial prescriptions may be caused by the differences in the original plant, doses, processing methods of Chinese medicines and extraction methods. Therefore, the plant origin, dose, processing method of medicines and extraction process should be regulated to obtain WDD preparations with consistent quality in each batch.

### Quantitative analysis of 11 components in WDD

According to this qualitative study, the primary bioactive constituents in WDD include flavonoids, triterpenoids, alkaloids, and phenolics. Then, naringin, hesperidin, neohesperidin, liquiritin, glycyrrhizic acid, adenosine, liquiritigenin, tangeretin, eriocitrin, naringenin and synephrine were quantified. The chromatograms of 4 compounds in positive ion MRM mode and 7 compounds in negative ion MRM mode are shown in Additional file 1: Figs. S16 and S17.

### Methodological verification

The calibration curve was constructed by diluting the sample solution into a specific gradient. Six concentration levels of standard stock solutions were diluted. The correlation coefficients (*r*) of the 11 components ranged from 0.9925 to 0.9994, indicating that the calibration curves were trustworthy for quantitative analysis. The limit of quantification (LOQ) for each component was determined by injecting a series of dilute solutions of known concentration at a signal-to-noise ratio (*S*/*N*) of 10. The LOQs ranged from 0.001 to 450.000 ng/mL (Additional file 1: Table S2).

The interday fluctuations, which were used to assess the precision of the devised approach, were studied by detecting the 11 analytes in 6 duplicates on a single day and repeating the tests. Six samples were prepared for the repeatability test. The sample solution was examined at various time periods (0, 2, 4, 8, 12, 24 h) to assess the sample's stability (Additional file 1: Table S3).

A recovery test was performed to confirm accuracy by introducing three concentration levels (low, medium, and high) of the mixed standard references into a certain quantity of sample solution. The recoveries ranged between 97.39% and 111.15%, with RSDs less than 8.86%, as detailed in Additional file 1: Table S4. All of these findings revealed that the developed procedures were sufficiently linear, precise, repeatable, stable, and recoverable for WDD sample quantification.

### Sample analysis

**Fig. 4:**
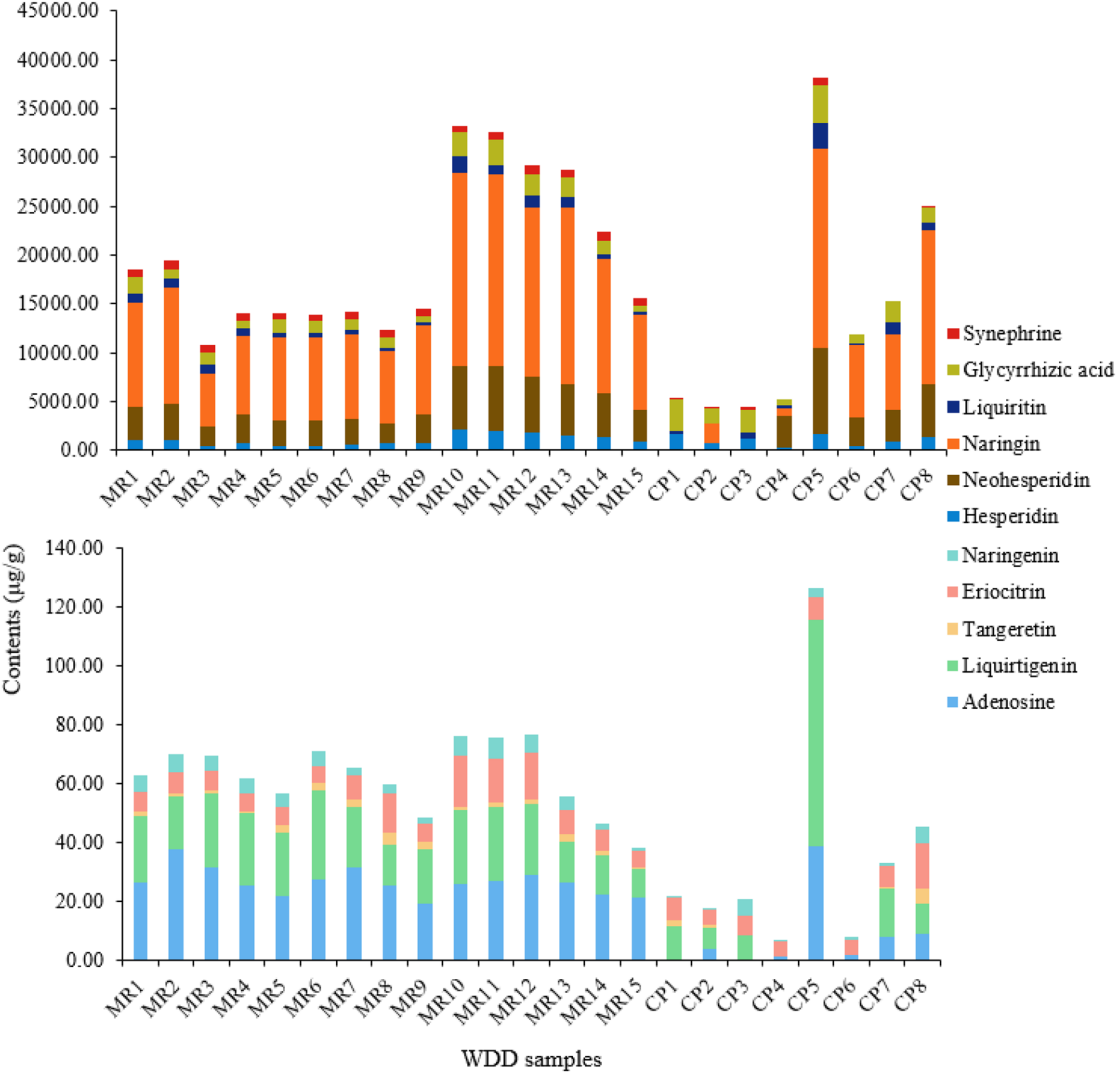
Contents of 11 representative components in the WDD material reference and its commercial preparations. (Due to the large difference in the content of 11 components, which do not fit in one vertical coordinate, they are divided into two parts. The figure above shows the contents of synephrine, glycyrrhizic acid, liquiritin, naringin, neohesperidin and hesperidin. The figure below shows the content of naringenin, eriocitrin, tangeretin, liquirtigenin and adenosine)

The established analytical approach was effectively used for the simultaneous determination of 11 typical components in the WDD material reference and its commercial preparations, as shown in Fig. 4 and Table 4. It was clear that naringin, neohesperidin and glycyrrhizic acid were the major constituents of the WDD material reference samples among the studied chemicals in the laboratory-made samples. Furthermore, naringin was the most abundant component in a daily dose. Modern research has shown that naringin and neohesperidin possess higher antioxidant and anti-inflammatory activities as well as effects on bone regeneration, metabolic syndrome, oxidative stress, genetic damage and central nervous system (CNS) diseases [52, 53]. Glycyrrhizic acid is the principal bioactive ingredient with antiviral, anti-inflammatory and hepatoprotective effects [54]. Therefore, these components play an important role in WDD in the treatment of cardiovascular diseases, hyperglycaemia and dyslipidaemia. The laboratory-made WDD material reference has relative standard deviation (RSD) values of contents in the range of 11.78–54.80%. However, commercial samples with RSD values in the range of 44.89–150.50% clearly showed significant differences in the concentrations of each identified component. In terms of the content of 11 components in the commercial preparation, only CP8 is close to the material reference by comparing the RSD 24.74–59.39%, which is in agreement with the statistical analysis of the heatmap. Other commercial preparations are more different from the material reference. In addition to liquirtigenin and glycyrrhizic acid of WDD, the contents of the other 9 compounds to be prepared in the laboratory were higher than those in commercial preparations. In summary, the contents of the components examined in the commercial WDD samples were highly diverse, indicating considerable variances in their quality.

**Table 4:** Contents of 11 representative components in the WDD material reference and its commercial preparations (μg/g, *n* = 3)

Type	Component	Adenosine	Synephrine	Liquirtigenin	Eriocitrin	Liquiritin	Naringenin	Naringin	Hesperidin	Neohesperidin	Glycyrrhizic acid	Tangeretin
Material reference	MR1	26.32 ± 6.24	778.62 ± 8.23	22.73 ± 9.62	6.63 ± 8.75	940.44 ± 7.89	5.64 ± 1.76	10645.14 ± 6.20	978.32 ± 9.04	3512.26 ± 9.61	1710.79 ± 8.24	1.63 ± 9.85
	MR2	37.68 ± 9.56	897.79 ± 9.65	17.96 ± 9.03	6.98 ± 9.58	852.53 ± 4.50	6.06 ± 4.67	11941.18 ± 3.13	1084.67 ± 9.82	3687.64 ± 5.16	982.64 ± 8.93	1.03 ± 9.61
	MR3	31.54 ± 9.18	814.52 ± 5.38	24.91 ± 5.40	6.81 ± 8.64	944.60 ± 6.19	5.38 ± 1.34	5452.55 ± 9.40	374.48 ± 6.28	2018.17 ± 9.27	1181.69 ± 3.90	0.97 ± 9.92
	MR4	25.25 ± 9.51	854.40 ± 4.86	24.85 ± 5.78	6.05 ± 8.96	853.63 ± 7.28	5.02 ± 1.95	8046.00 ± 9.87	686.94 ± 8.10	2934.56 ± 9.77	701.07 ± 5.05	0.58 ± 9.64
	MR5	21.73 ± 9.40	595.87 ± 9.29	21.66 ± 9.69	5.91 ± 8.02	556.13 ± 7.54	4.82 ± 7.74	8490.68 ± 6.31	478.75 ± 9.53	2565.42 ± 5.28	1286.23 ± 4.31	2.59 ± 8.49
	MR6	27.37 ± 7.79	651.39 ± 1.99	30.18 ± 9.72	5.67 ± 7.02	446.18 ± 8.41	4.82 ± 7.74	8490.68 ± 6.31	478.75 ± 9.53	2565.42 ± 5.28	1259.08 ± 4.31	2.77 ± 8.49
	MR7	31.46 ± 7.24	634.51 ± 9.88	20.72 ± 7.13	8.18 ± 9.36	455.35 ± 6.84	2.68 ± 9.34	8541.90 ± 9.51	629.97 ± 9.92	2641.52 ± 6.76	1214.65 ± 8.67	2.33 ± 9.13
	MR8	25.21 ± 9.47	750.37 ± 6.11	13.97 ± 8.87	13.49 ± 9.80	428.60 ± 4.30	3.15 ± 9.69	7405.05 ± 9.01	676.53 ± 9.70	2020.22 ± 8.44	1101.30 ± 9.31	3.87 ± 9.85
	MR9	19.27 ± 7.98	854.40 ± 6.16	18.42 ± 8.76	6.39 ± 8.42	431.26 ± 4.66	2.05 ± 8.16	9073.31 ± 5.13	764.35 ± 8.39	2892.98 ± 6.78	553.54 ± 6.78	2.43 ± 8.30
	MR10	25.92 ± 9.14	666.82 ± 2.95	24.84 ± 6.06	17.05 ± 8.33	1683.29 ± 8.35	6.75 ± 8.15	19814.33 ± 3.75	2150.99 ± 5.38	6442.32 ± 4.58	2469.72 ± 4.70	1.44 ± 8.39
	MR11	26.90 ± 5.02	678.86 ± 9.20	25.21 ± 3.45	15.03 ± 8.24	955.20 ± 7.86	6.83 ± 3.68	19690.75 ± 6.61	2027.77 ± 3.13	6542.79 ± 2.46	2645.80 ± 2.46	1.32 ± 718
	MR12	28.84 ± 7.53	783.21 ± 7.54	24.34 ± 6.90	15.50 ± 5.97	1213.88 ± 1.24	6.38 ± 7.41	17382.59 ± 6.69	1786.87 ± 9.62	5731.72 ± 6.73	2215.76 ± 5.21	1.53 ± 4.01
	MR13	26.54 ± 9.69	787.23 ± 2.34	13.87 ± 8.80	8.04 ± 7.49	1115.40 ± 8.43	4.74 ± 9.06	18122.54 ± 9.88	1446.26 ± 9.37	5297.31 ± 9.54	1970.65 ± 8.66	2.33 ± 9.94
	MR14	22.35 ± 8.91	849.69 ± 2.71	13.12 ± 6.78	7.52 ± 9.18	574.23 ± 9.60	2.05 ± 9.26	13742.52 ± 6.01	1302.74 ± 1.90	4513.54 ± 1.04	1325.38 ± 4.07	1.52 ± 9.94
	MR15	21.13 ± 9.11	757.98 ± 5.71	9.67 ± 8.64	5.52 ± 8.93	292.25 ± 9.63	1.00 ± 9.78	9695.17 ± 9.63	850.53 ± 8.59	3309.01 ± 7.41	714.09 ± 8.27	0.89 ± 9.78
	AVG	26.50	749.39	20.43	8.98	782.86	4.49	11768.96	1047.86	3778.33	1422.16	1.82
	RSD (%)	17.64	11.78	28.16	45.11	48.32	41.45	40.68	54.80	41.02	45.40	48.73
Commercial preparations	CP1	0.016 ± 5.17	143.53 ± 9.86	11.33 ± 9.16	7.36 ± 8.96	257.59 ± 7.22	0.84 ± 8.77	ND	1649.58 ± 8.83	ND	3334.92 ± 8.61	2.48 ± 6.28
	CP2	4.08 ± 4.55	51.01 ± 6.04	6.85 ± 9.02	5.28 ± 7.39	106.52 ± 9.97	0.27 ± 7.77	2027.77 ± 9.63	680.69 ± 7.17	ND	1424.23 ± 5.74	1.08 ± 7.60
	CP3	ND	366.32 ± 8.23	8.74 ± 1.37	6.58 ± 3.55	533.87 ± 2.34	5.64 ± 4.44	ND	1216.69 ± 4.93	ND	2309.10 ± 6.02	ND
	CP4	1.52 ± 7.86	ND	ND	5.12 ± 8.36	155.13 ± 7.83	0.53 ± 8.56	888.34 ± 9.22	237.94 ± 3.06	3236.89 ± 5.21	721.03 ± 8.80	ND
	CP5	38.56 ± 8.45	738.06 ± 7.90	76.77 ± 7.36	7.70 ± 8.58	2568.08 ± 4.69	3.15 ± 9.47	20406.24 ± 3.19	1719.75 ± 3.94	8810.34 ± 2.20	3915.97 ± 2.84	0.020 ± 9.20
	CP6	1.70 ± 9.02	ND	ND	5.23 ± 5.20	121.15 ± 9.97	0.99 ± 7.60	7456.44 ± 4.29	496.46 ± 4.04	2897.91 ± 4.11	959.36 ± 5.74	ND
	CP7	7.78 ± 9.25	ND	16.68 ± 7.56	7.34 ± 8.53	1251.99 ± 9.28	1.04 ± 9.69	7700.01 ± 8.31	957.48 ± 7.88	3236.89 ± 5.21	2193.21 ± 8.71	0.28 ± 5.70
	CP8	9.02 ± 5.75	119.13 ± 6.85	10.04 ± 8.61	15.42 ± 9.01	880.37 ± 7.96	5.64 ± 5.93	15756.12 ± 9.63	1280.16 ± 9.37	5432.06 ± 8.56	1500.00 ± 6.39	5.21 ± 4.49
	AVG	8.95	283.61	21.73	7.50	734.34	2.26	9039.15	1029.84	4722.82	204.73	1.81
	RSD (%)	150.50	98.81	124.99	44.89	115.38	99.86	84.86	51.86	52.89	55.05	117.09
CP8-material reference^a^	RSD (%)	24.74	25.23	31.01	45.09	46.42	39.91	39.37	52.50	40.01	43.73	59.39

MR1-MR15 represent 15 batches of WDD material reference prepared in the laboratory; CP1-CP8 are commercial preparations of WDD

*AVG* Average, *ND* Not detectable

^a^ RSDs of CP8 and 15 batches of material reference were compared

## Conclusions

In this study, UPLC-QTOF-MS technology combined with UNIFI software was used for the first time to characterize the chemical composition of the WDD material reference. The performed research showed that the established method could quickly identify the chemical composition of WDD and quickly evaluate the difference between the WDD material reference and its commercial prescriptions. A total of 107 compounds were identified, including 54 flavonoids, 14 triterpenes, 10 organic acids, 7 alkaloids, 7 coumarins and 15 other components. The fragmentation patterns of representative compounds in each category were deduced. In the heatmap and PCA, it was found that the WDD material reference and its commercial prescriptions were divided into one second-level cluster, which indicated the diversity of different preparation methods. Luckily, an obvious tendency of accumulation of CP8 in the WDD material reference and its commercial prescriptions was discovered.

Eleven representative compounds were selected, and the content determination method was established to compare the content difference between the WDD material reference and its commercial prescriptions. The quality of commercial formulas also varies. The 11 compounds showed that the content of the WDD material reference was generally higher than that of its commercial prescriptions. The performed research showed that the established method could quickly identify the chemical composition of WDD and quickly evaluate the difference between the WDD material reference and its commercial prescriptions. In addition, only one batch of CP8 in commercial prescriptions was close to the reference material. It is speculated that the choice of plant origin and preparation method of CP8 would be consistent with the WDD material reference. In summary, this paper compared the WDD material reference and its commercial prescriptions based on UPLC-QTOF-MS and multivariate statistical analysis. The quality of most WDD commercial prescriptions was unstable and significantly different from the WDD material reference, which could impact the effectiveness and safety in the clinic. Therefore, to ensure the uniformity and stability of the efficacy and quality of the formula, further studies should be conducted based on material references providing data support for the research and development of the ancient classical Chinese medicinal formulas.

## Supplementary Information

**Additional file 1: Table S1.** Mass spectrometer parameters for MRM of analytes. **Table S2.** Calibration curves, correlation coefficient (r) and linear ranges of 11 analytes. **Table S3.** Precision, repeatability and stability of 11 analytes (n = 3). **Table S4.** Recoveries of 11 representative components in the WDD (n = 3). **Figure S1.** Base peak intensity (BPI) chromatograms of Pinelliae Rhizoma Praeparatum in negative (A) and positive (B) ion modes. **Figure S2.** Base peak intensity (BPI) chromatograms of Bambusae Caulis in Taenias in negative (A) and positive (B) ion modes. **Figure S3.** Base peak intensity (BPI) chromatograms of Aurantii Fructus Immaturus in negative (A) and positive (B) ion modes. **Figure S4.** Base peak intensity (BPI) chromatograms of Citri Reticulatae Pericarpium in negative (A) and positive (B) ion modes. **Figure S5.** Base peak intensity (BPI) chromatograms of Glycyrrhizae Radix et Rhizoma in negative (A) and positive (B) ion modes. **Figure S6.** Base peak intensity (BPI) chromatograms of Zingiberis Rhizoma Recens in negative (A) and positive (B) ion modes. **Figure S7.** The mass spectrum and fragmentation pathways of neohesperidin in positive ion mode. **Figure S8.** The mass spectrum and fragmentation pathways of diosmetin 6,8-di-C-glucoside in negative ion mode. **Figure S9.** The mass spectrum and fragmentation pathways of 3,5,6,7,8,3′,4′-heptamethoxyflavone in positive ion mode. **Figure S10.** The mass spectrum and fragmentation pathways of glycyrrhizic acid in positive ion mode. **Figure S11.** The mass spectrum and fragmentation pathways of obacunone in positive ion mode. **Figure S12.** The mass spectrum and fragmentation pathways of 3,4-dihydroxybenzoic acid in positive ion mode. **Figure S13.** The mass spectrum and fragmentation pathways of synephrine in positive ion mode. **Figure S14.** The mass spectrum and fragmentation pathways of meranzin in positive ion mode. **Figure S15.** The mass spectrum and fragmentation pathways of 6-gingerol in positive ion mode. **Figure S16.** Chromatograms of 4 compounds in positive ion MRM mode. (A) Adenosine; (B) Liquirtigenin; (C) Synephrine; (D) Tangeretin. **Figure S17.** Chromatograms of 7 compounds in negative ion MRM mode. (A) Eriocitrin; (B) Naringenin; (C) Neohesperidin; (D) Hesperidin; (E) Naringin; (F) Liquiritin; (G) Glycyrrhizic acid.

## Data Availability

The datasets used and analysed during the current study are available from the corresponding author on reasonable request.

## Abbreviations

AFI: Aurantii Fructus Immaturus
BCT: Bambusae Caulis in Taenias
BPI: Base peak intensity
COVID-19: Coronavirus disease 2019
CRP: Citri Reticulatae Pericarpium
GRR: Glycyrrhizae Radix et Rhizoma
LOQ: Limit of quantification
MRM: Multiple reaction monitoring
PCA: Principal component analysis
PR: Pinelliae Rhizoma
*r*: Correlation coefficients
RSD: Relative standard deviation
S/N: Signal-to-noise ratio
TCM: Traditional Chinese medicine
WDD: Wendan decoction
ZRR: Zingiberis Rhizoma Recens
