# Supplementary material for: UPLC-QTOF-MS with a chemical profiling approach for holistic quality evaluation between a material reference of Wen Dan decoction and its commercial preparations
Source: Chin Med. 2023 May 30;18:63. doi: 10.1186/s13020-023-00767-z (PMC10226876; doi:10.1186/s13020-023-00767-z)
Supplement: Supplementary file 1 — Additional file 1: Table S1. Mass spectrometer parameters for MRM of analytes. Table S2. Calibration curves, correlation coefficient (r) and linear ranges of 11 analytes. Table S3. Precision, repeatability and stability of 11 analytes (n = 3). Table S4. Recoveries of 11 representative components in the WDD (n = 3). Figure S1. Base peak intensity (BPI) chromatograms of Pinelliae Rhizoma Praeparatum in negative (A) and positive (B) ion modes. Figure S2. Base peak intensity (BPI) chromatograms of Bambusae Caulis in Taenias in negative (A) and positive (B) ion modes. Figure S3. Base peak intensity (BPI) chromatograms of Aurantii Fructus Immaturus in negative (A) and positive (B) ion modes. Figure S4. Base peak intensity (BPI) chromatograms of Citri Reticulatae Pericarpium in negative (A) and positive (B) ion modes. Figure S5. Base peak intensity (BPI) chromatograms of Glycyrrhizae Radix et Rhizoma in negative (A) and positive (B) ion modes. Figure S6. Base peak intensity (BPI) chromatograms of Zingiberis Rhizoma Recens in negative (A) and positive (B) ion modes. Figure S7. The mass spectrum and fragmentation pathways of neohesperidin in positive ion mode. Figure S8. The mass spectrum and fragmentation pathways of diosmetin 6,8-di-C-glucoside in negative ion mode. Figure S9. The mass spectrum and fragmentation pathways of 3,5,6,7,8,3′,4′-heptamethoxyflavone in positive ion mode. Figure S10. The mass spectrum and fragmentation pathways of glycyrrhizic acid in positive ion mode. Figure S11. The mass spectrum and fragmentation pathways of obacunone in positive ion mode. Figure S12. The mass spectrum and fragmentation pathways of 3,4-dihydroxybenzoic acid in positive ion mode. Figure S13. The mass spectrum and fragmentation pathways of synephrine in positive ion mode. Figure S14. The mass spectrum and fragmentation pathways of meranzin in positive ion mode. Figure S15. The mass spectrum and fragmentation pathways of 6-gingerol in positive ion mode. Figure S16. Chromatograms [file 13020_2023_767_MOESM1_ESM.doc]

**Supplementary information**

**UPLC-QTOF-MS with a chemical profiling approach for** **holistic quality evaluation between a material reference of Wen Dan decoction and** **its commercial preparations**

Siyu Yang^1,2^, Gan Chen^1,2^, Man Yuan^1,2^, Yan Zou^3^, Hongmei Zhang1,[[1]](#footnote-2), Hongxi Xu^4^,**

^1^ School of Pharmacy, Shanghai University of Traditional Chinese Medicine, Shanghai 201203, P.R. China

^2^ Engineering Research Center of Shanghai Colleges for TCM New Drug Discovery, Shanghai 201203, P.R. China

^3^ Shineway Pharmaceutical Group Ltd., Hebei, China

^4^ Shuguang Hospital, Shanghai University of Traditional Chinese Medicine, Shanghai 201203, P.R. China


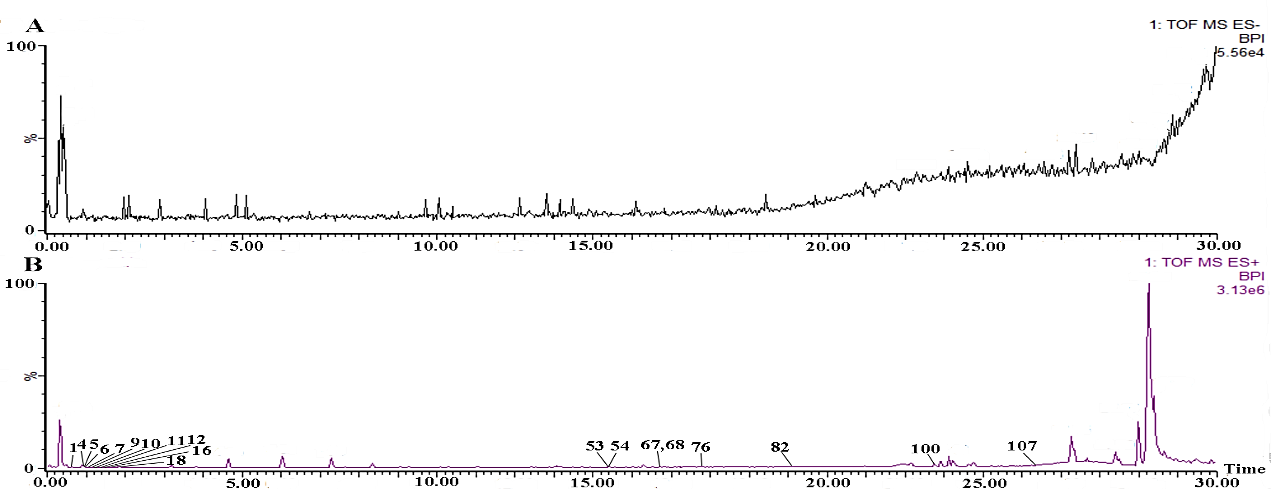

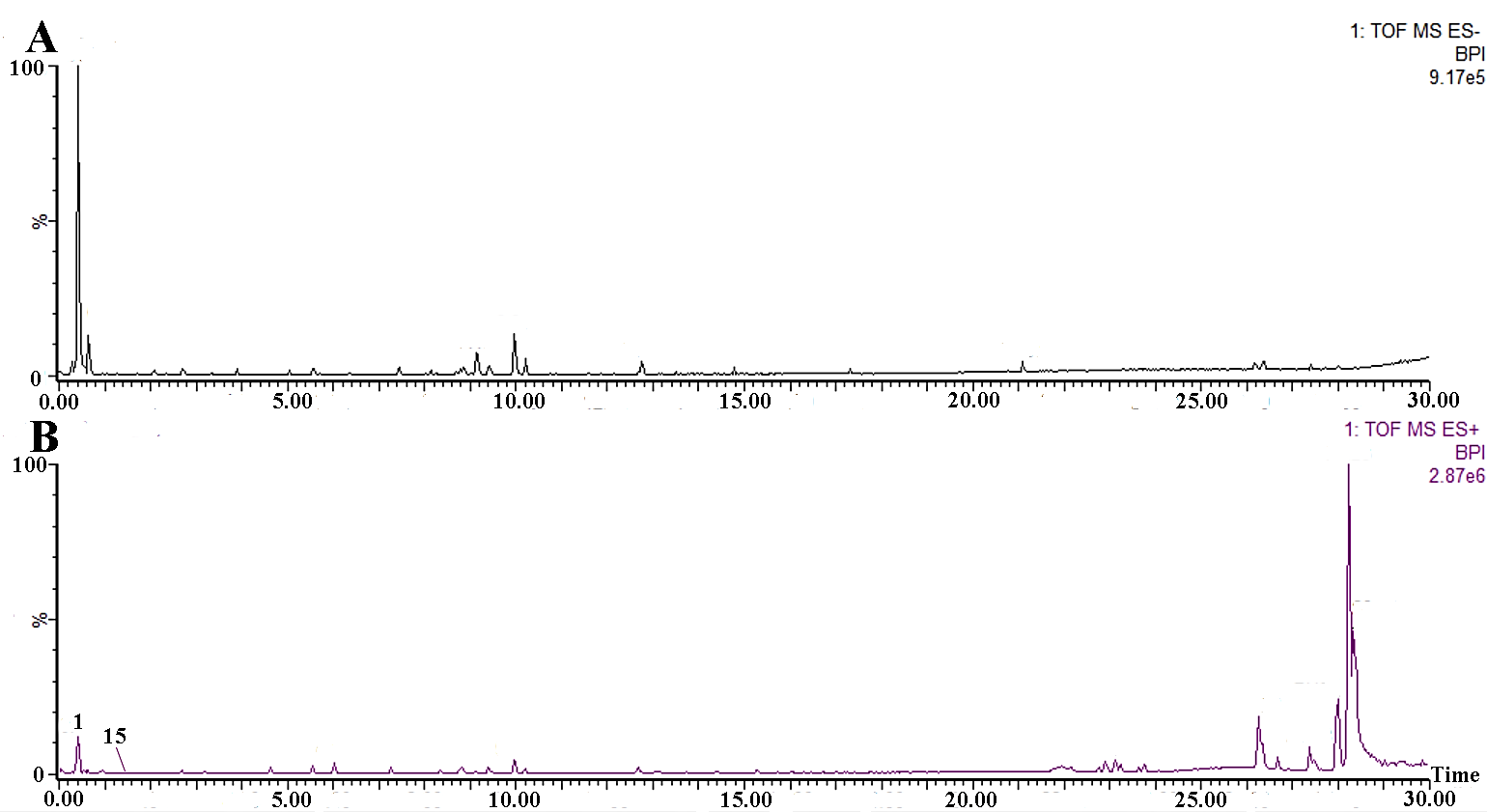
**Fig. S1.** Base peak intensity (BPI) chromatograms of Pinelliae Rhizoma Praeparatum in negative (A) and positive (B) ion modes.

**Fig. S2.** Base peak intensity (BPI) chromatograms of Bambusae Caulis in Taenias in negative (A) and positive (B) ion modes.


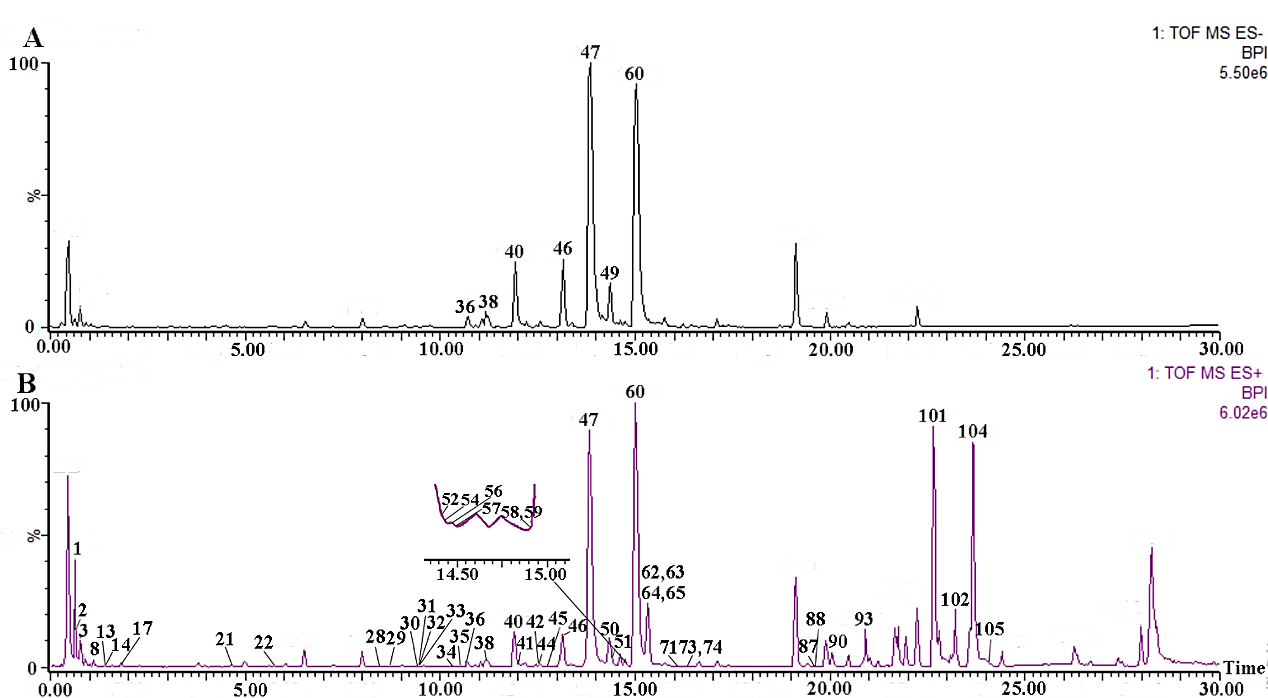
**Fig. S3.** Base peak intensity (BPI) chromatograms of Aurantii Fructus Immaturus in negative (A) and positive (B) ion modes.


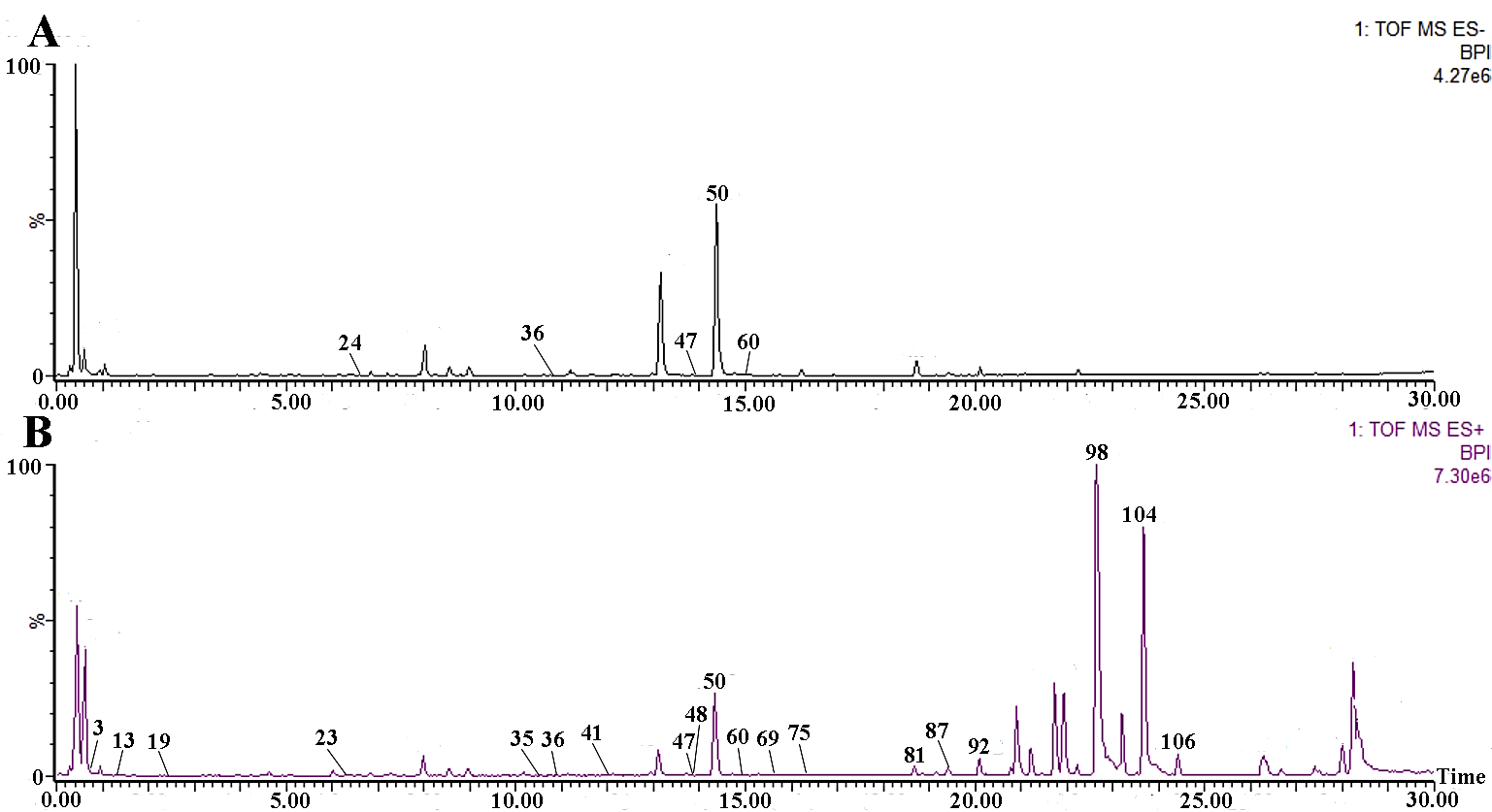


**Fig. S4.** Base peak intensity (BPI) chromatograms of Citri Reticulatae Pericarpium in negative (A) and positive (B) ion modes.


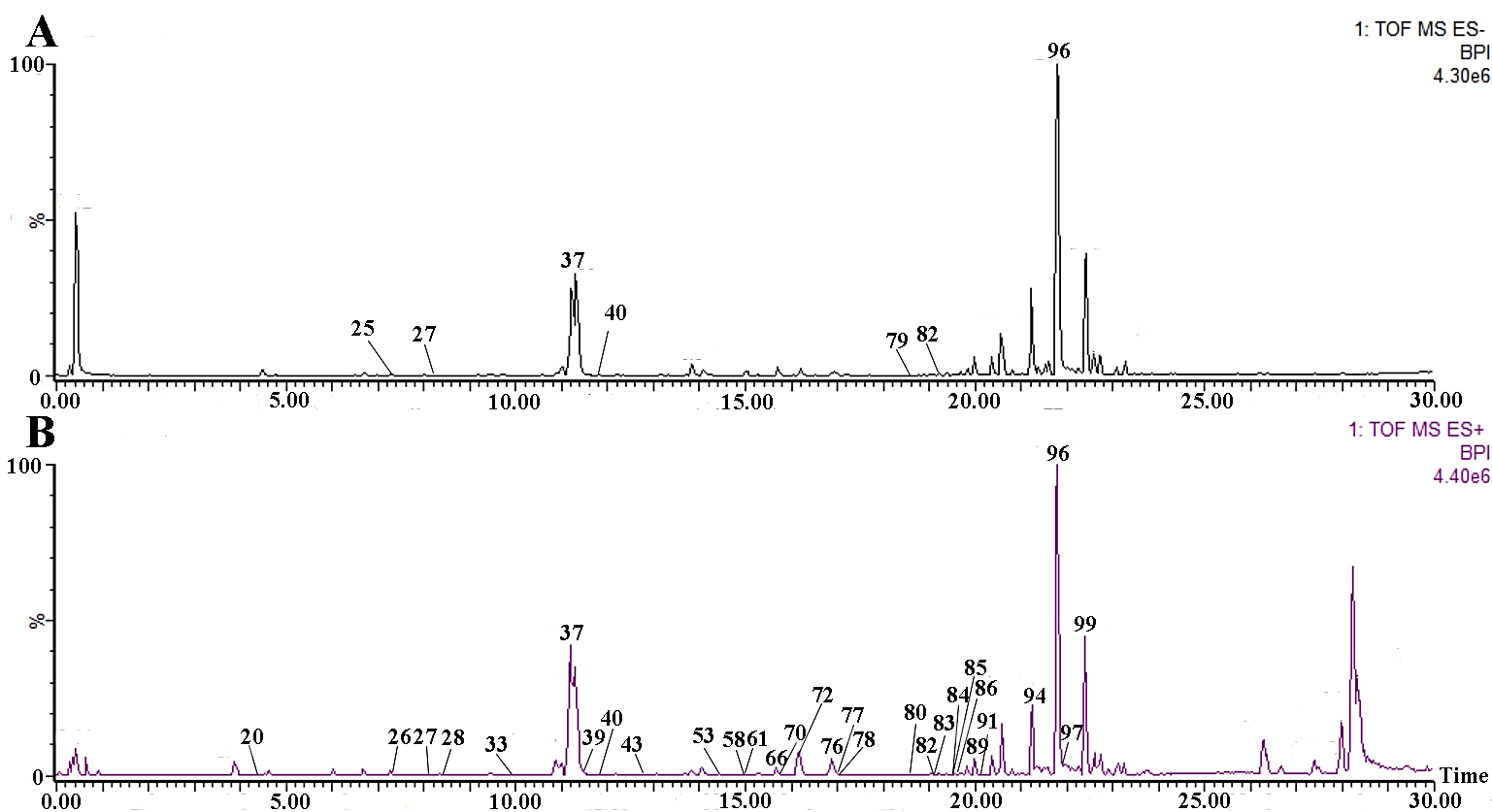
**Fig. S5.** Base peak intensity (BPI) chromatograms of Glycyrrhizae Radix et Rhizoma in negative (A) and positive (B) ion modes.


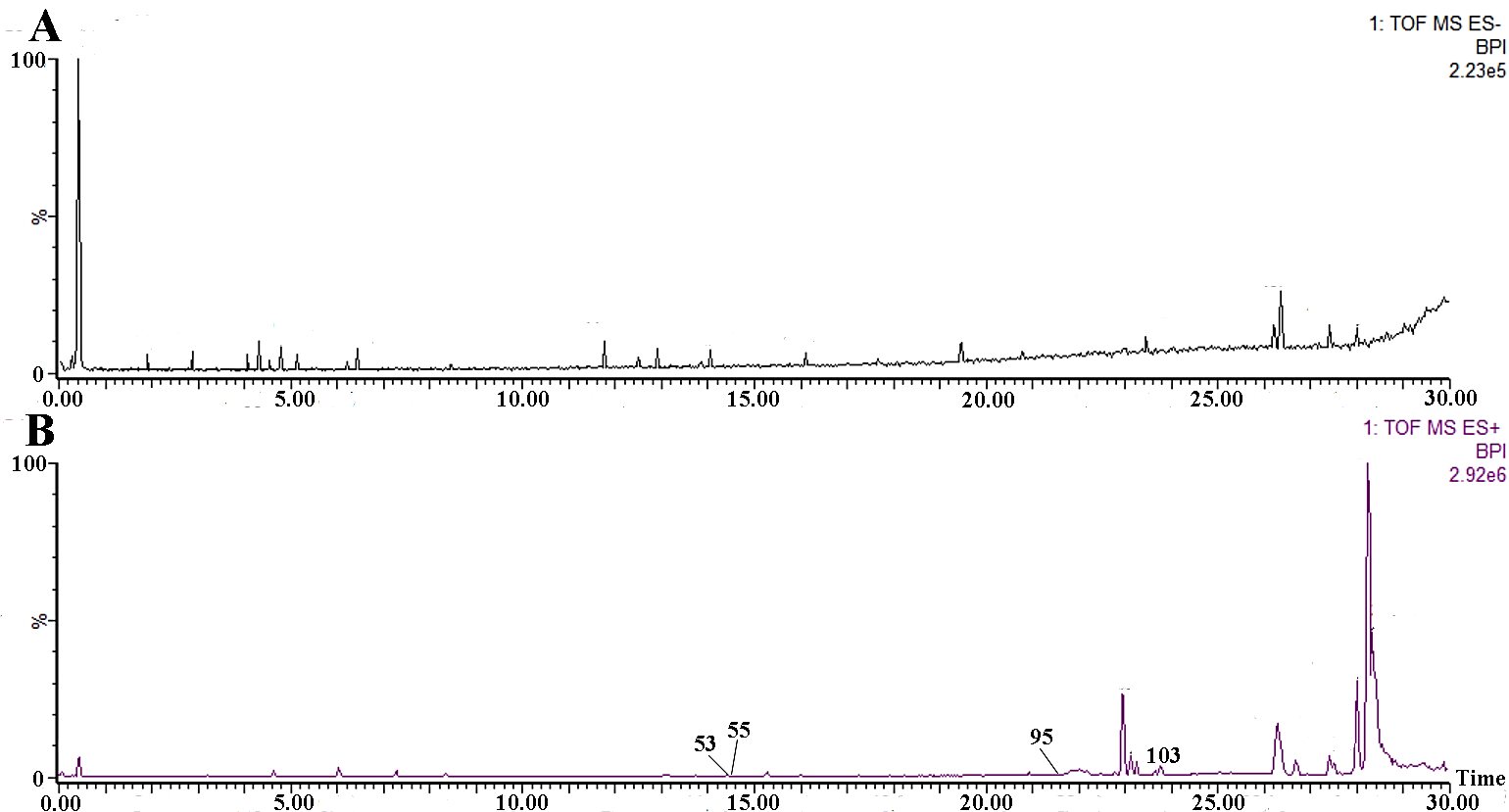


**Fig. S6.** Base peak intensity (BPI) chromatograms of Zingiberis Rhizoma Recens in negative (A) and positive (B) ion modes.


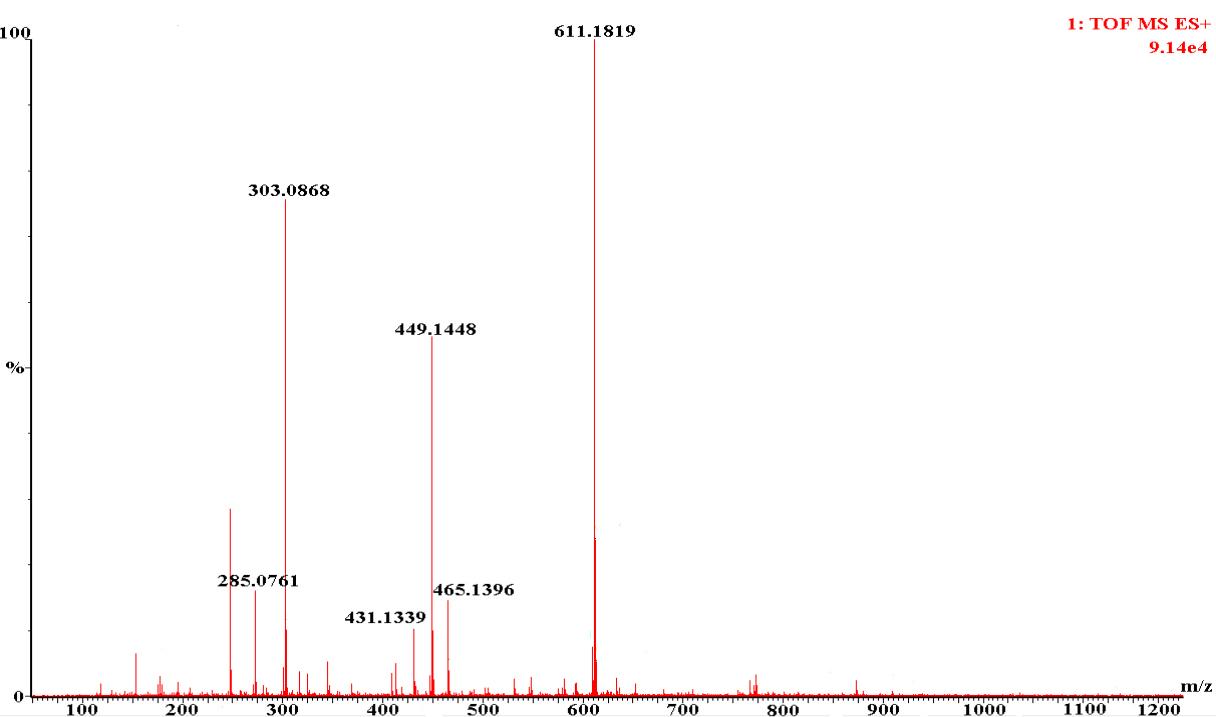


**Fig. S7.** The mass spectrogram and fragmentation pathways of neohesperidin in positive ion mode.


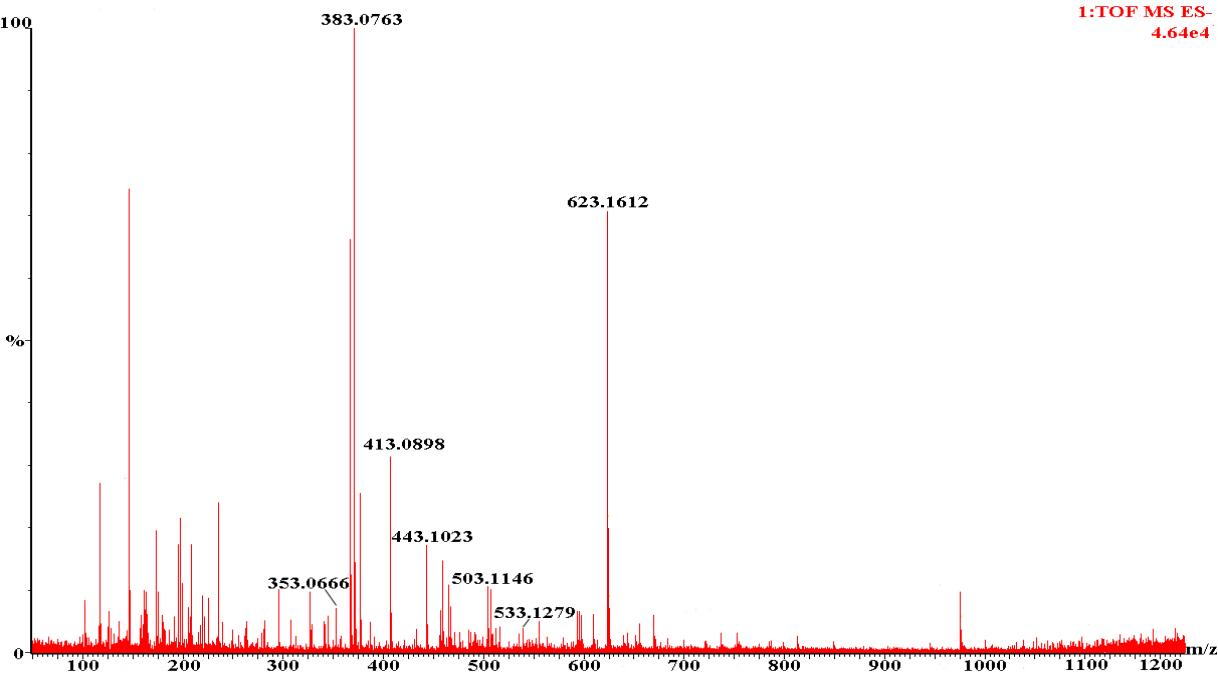


**Fig. S8.** The mass spectrogram and fragmentation pathways of diosmetin 6,8-di-C-glucoside in negative ion mode.


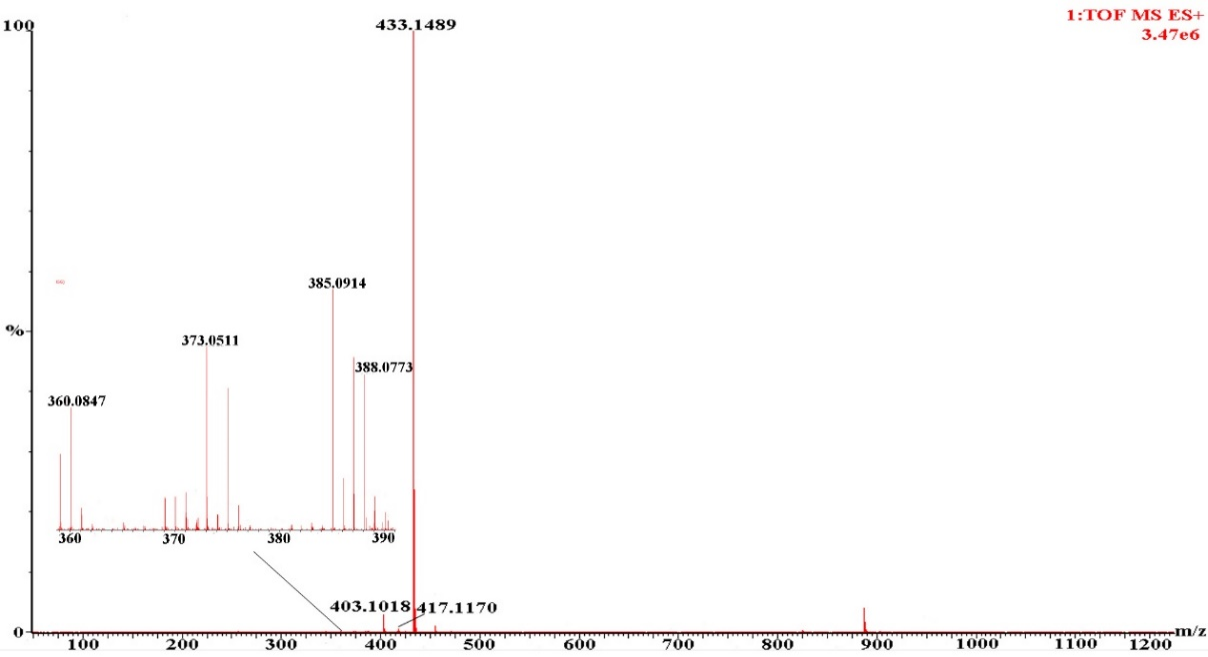


**Fig. S9.** The mass spectrogram and fragmentation pathways of 3,5,6,7,8,3′,4′-heptamethoxyflavone in positive ion mode.


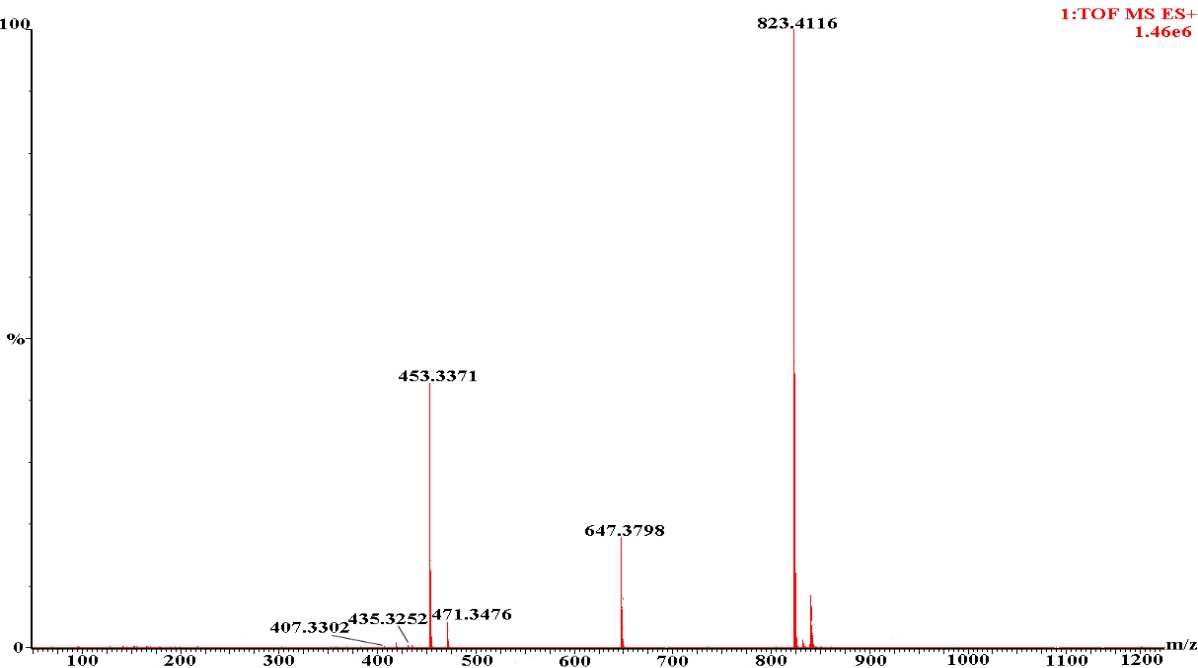


**Fig. S10.** The mass spectrogram and fragmentation pathways of glycyrrhizic acid in positive ion mode.


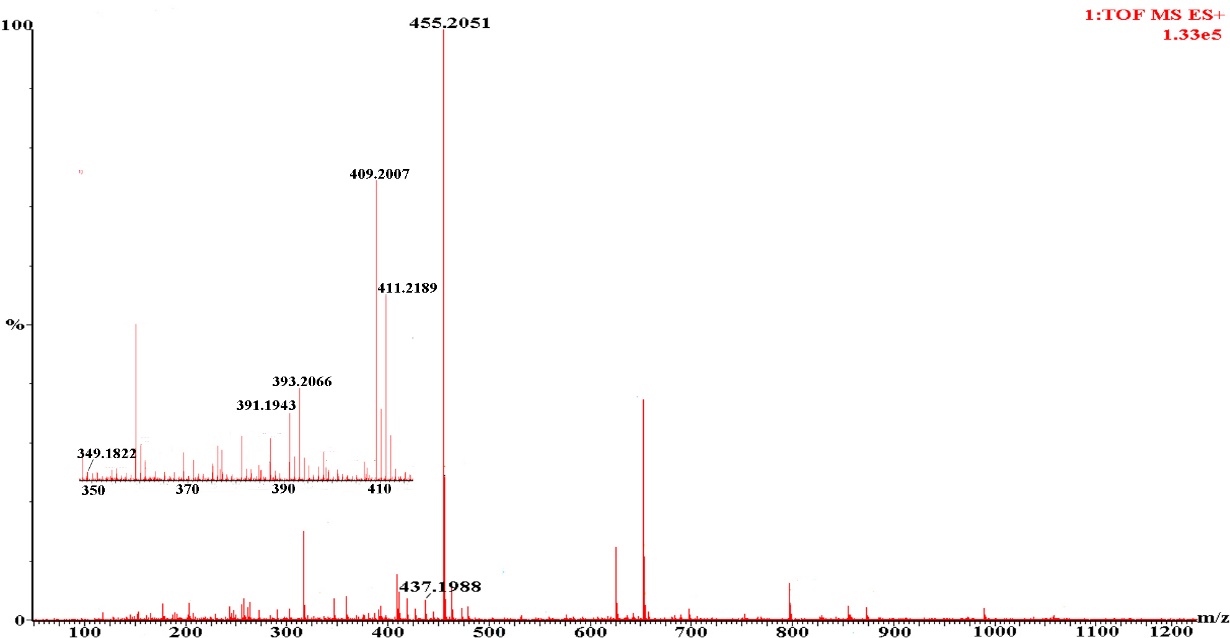


**Fig. S11.** The mass spectrogram and fragmentation pathways of obacunone in positive ion mode.


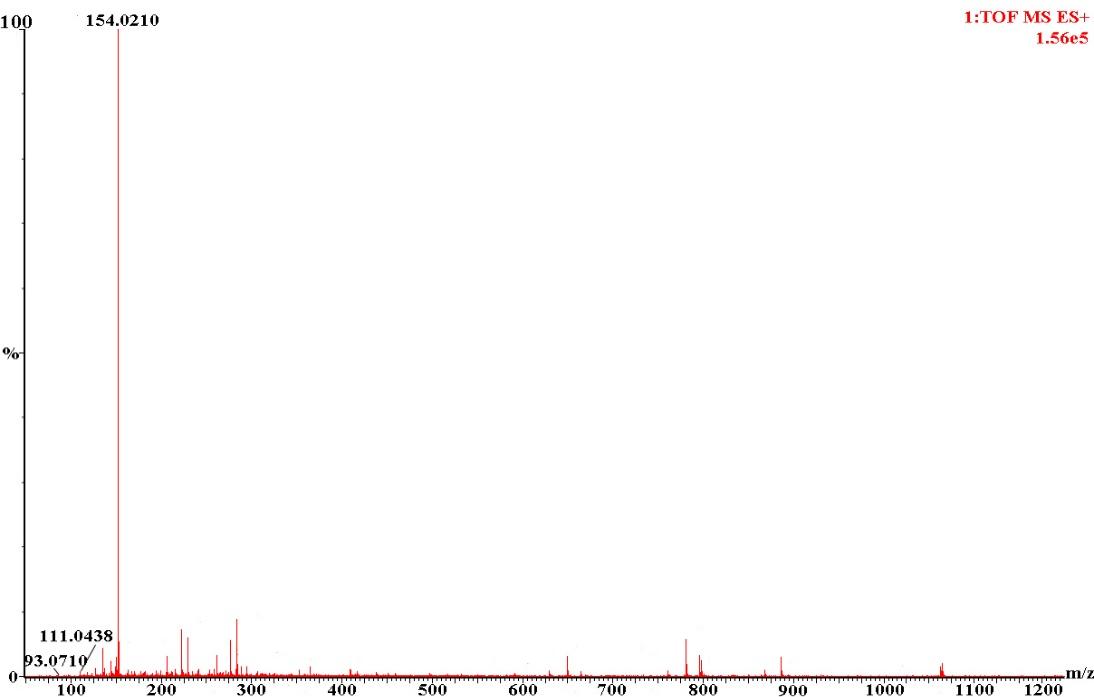


**Fig. S12.** The mass spectrogram and fragmentation pathways of 3,4-dihydroxybenzoic acid in positive ion mode.


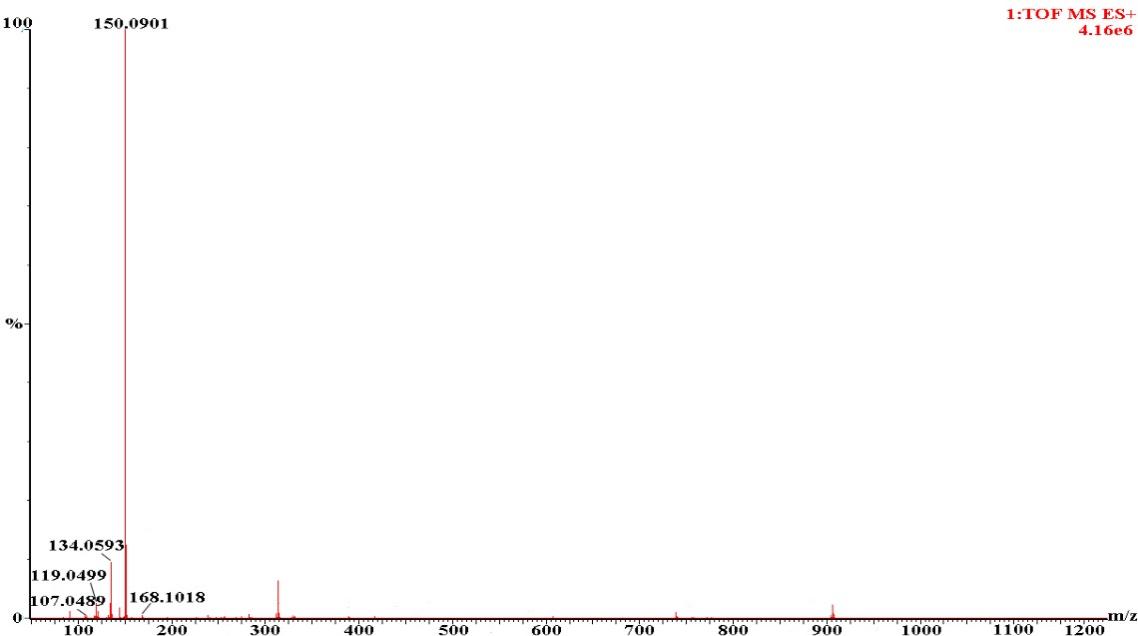


**Fig. S13.** The mass spectrogram and fragmentation pathways of synephrine in positive ion mode.


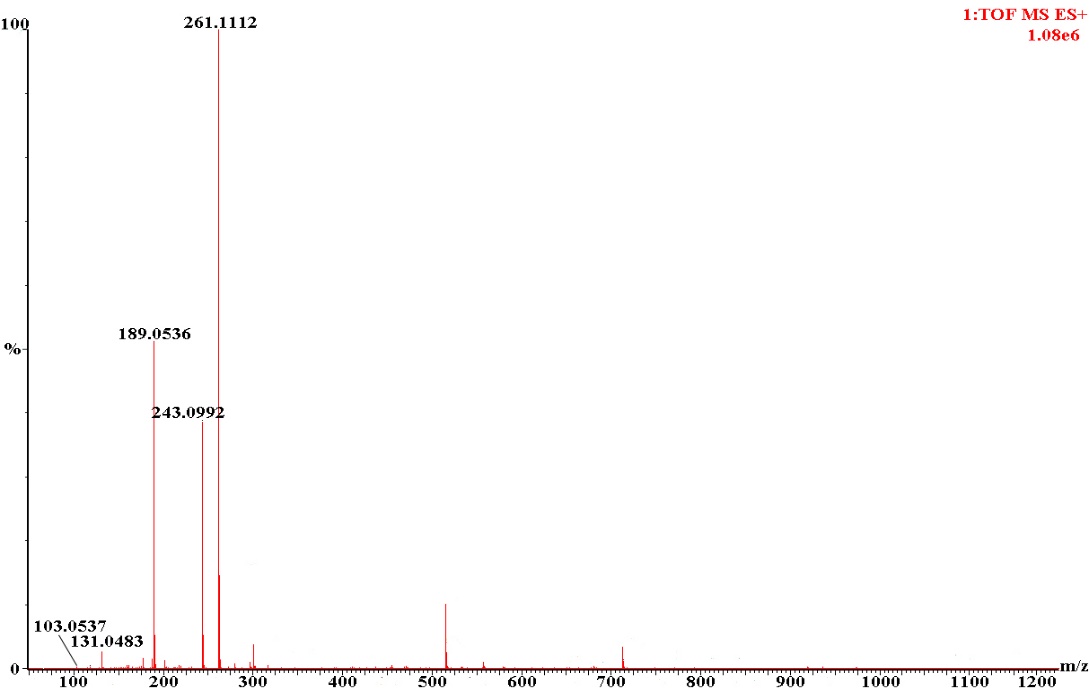


**Fig. S14.** The mass spectrogram and fragmentation pathways of meranzin in positive ion mode


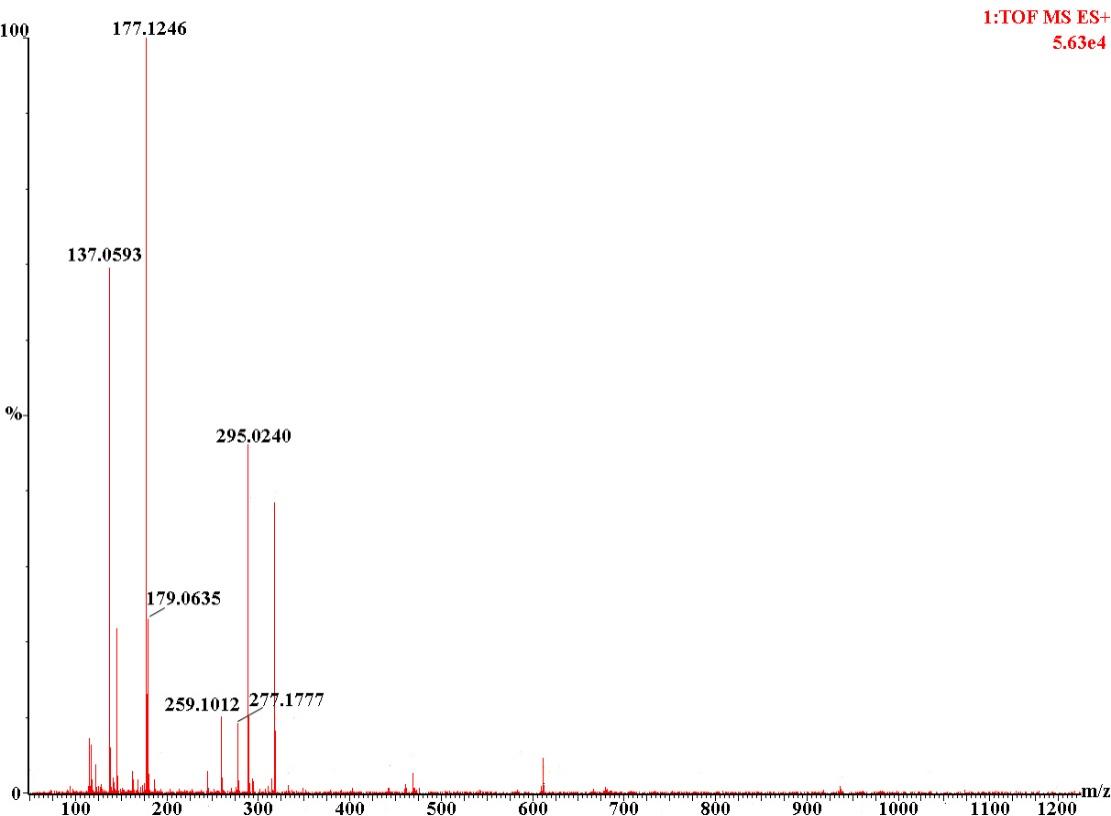


**Fig. S15.** The mass spectrogram and fragmentation pathways of 6-gingerol in positive ion mode.


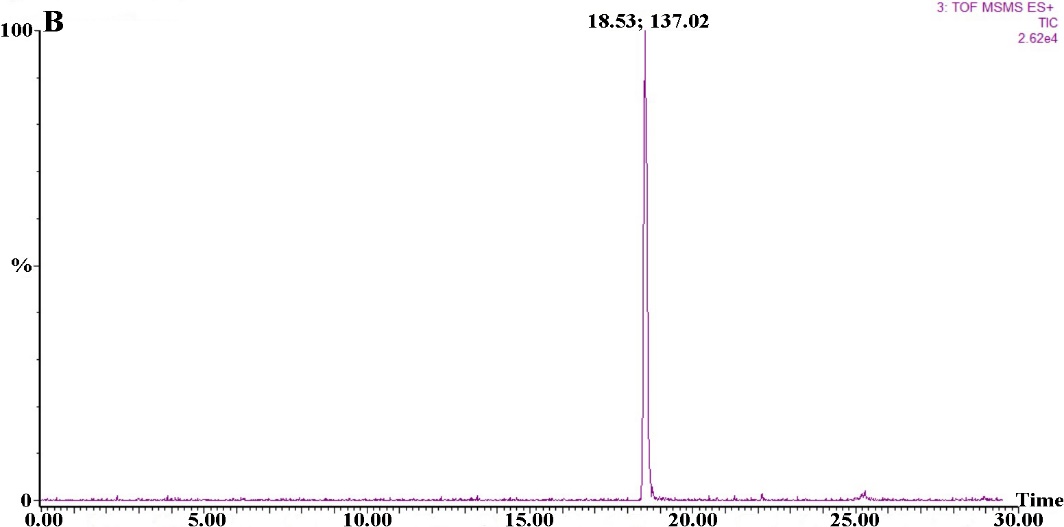

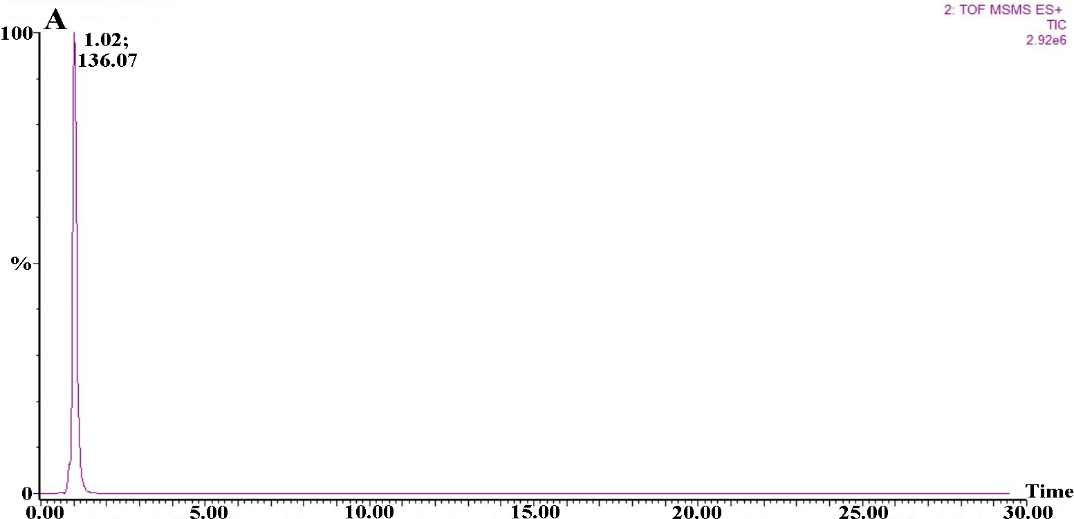

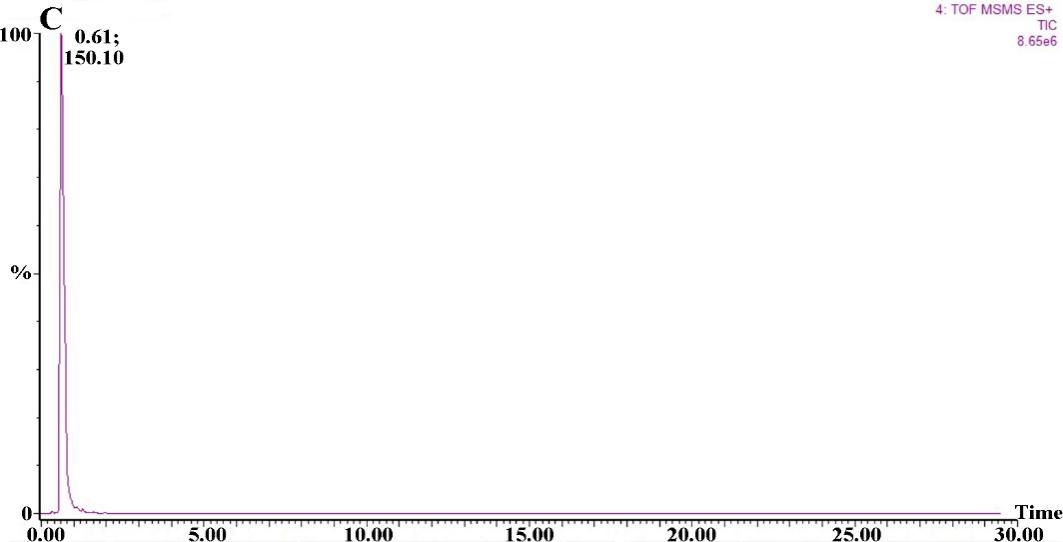

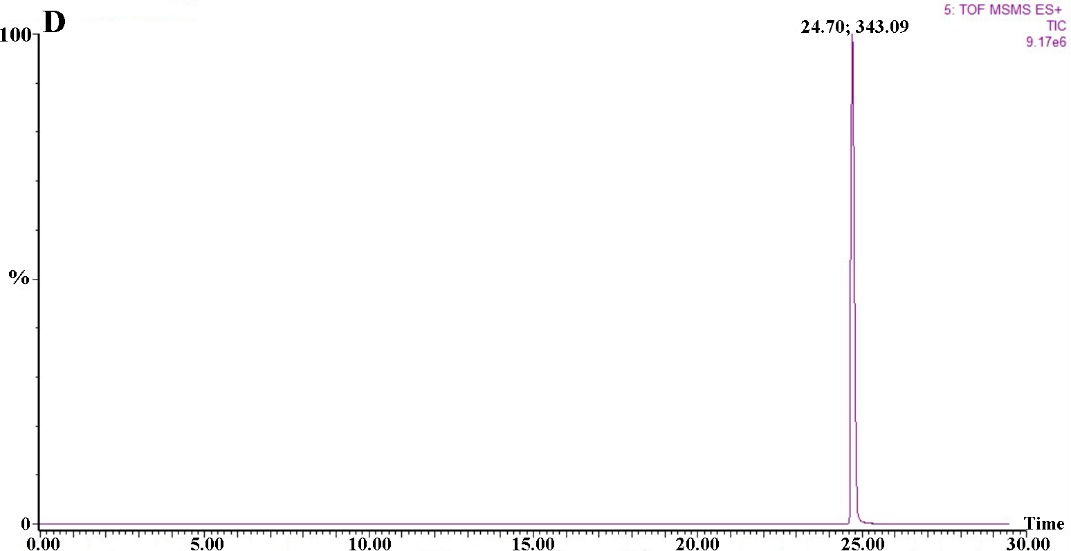
**Fig. S16.** Chromatograms of 4 compounds in positive ion MRM mode. (A) Adenosine; (B) Liquirtigenin; (C) Synephrine; (D) Tangeretin.


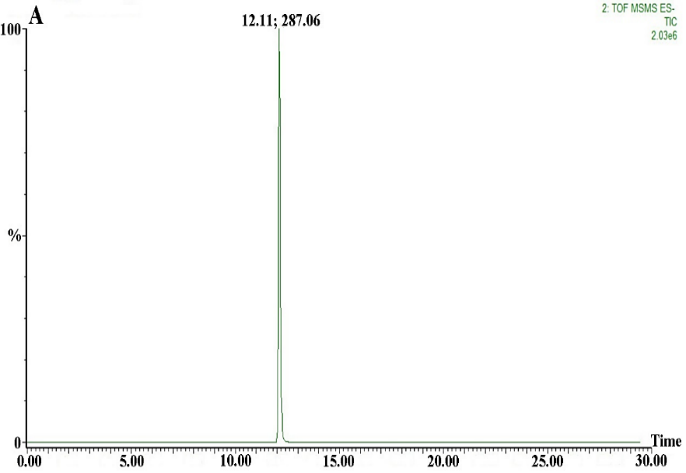

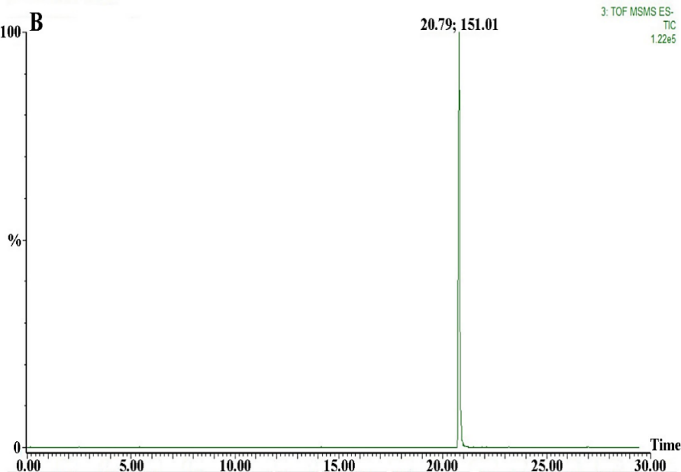

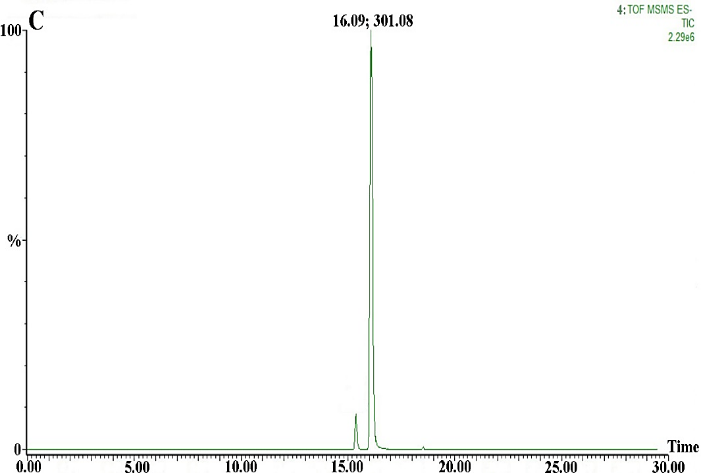

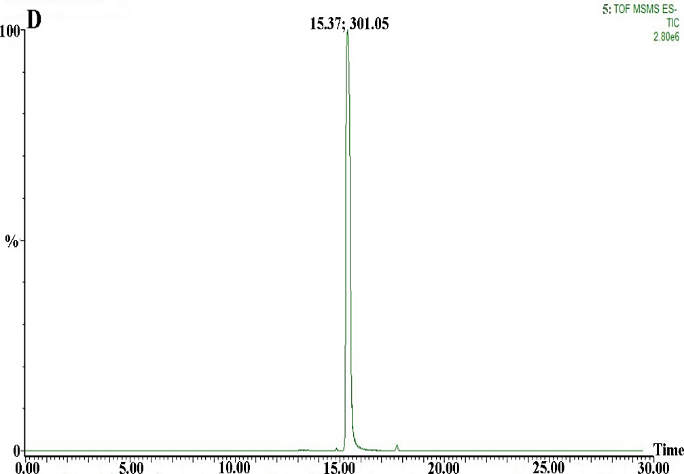

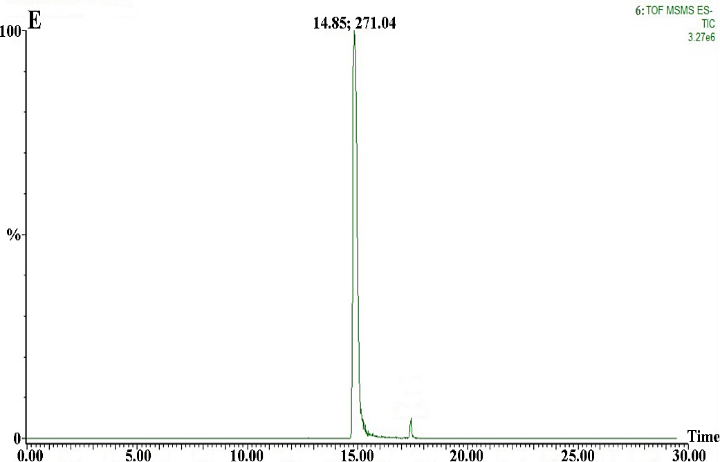

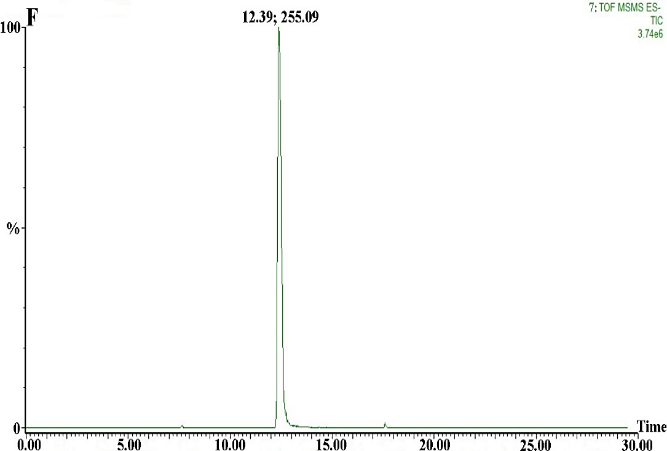

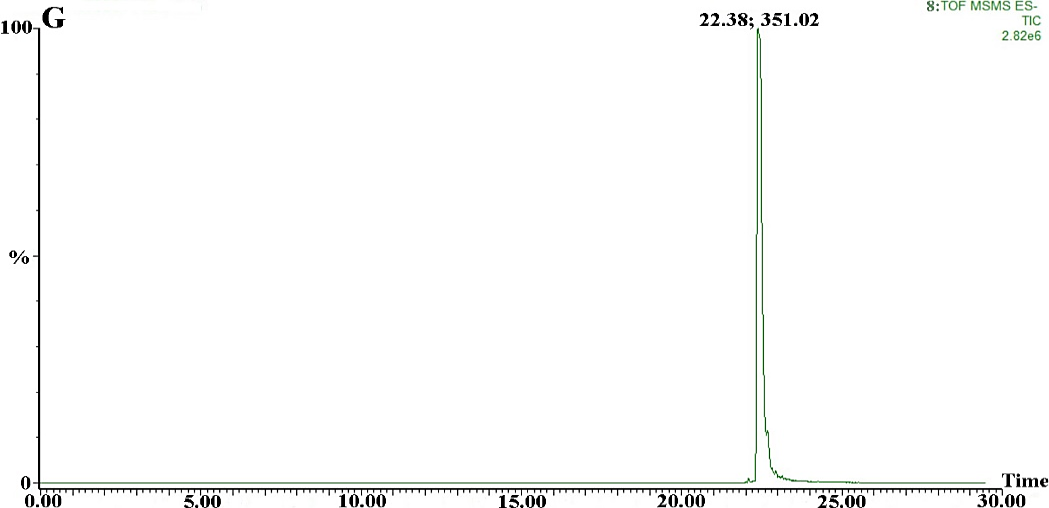


**Fig. S17.** Chromatograms of 7 compounds in negative ion MRM mode. (A) Eriocitrin; (B) Naringenin; (C) Neohesperidin; (D) Hesperidin; (E) Naringin; (F) Liquiritin; (G) Glycyrrhizic acid.

**Table S1 Mass spectrometer parameters for MRM of analytes.**

| Compounds | Molecule Weight | MRM transition | Cone (V) | Collision (V) | *t*_R_ (min) | Ion mode |
| --- | --- | --- | --- | --- | --- | --- |
| Adenosine | 267.24 | 268.11→136.10 | 10 | 10 | 0.63 | ESI^+^ |
| Synephrine | 167.21 | 150.14→135.12 | 45 | 10 | 0.73 | ESI^+^ |
| Liquirtigenin | 256.25 | 257.12→137.07 | 10 | 5 | 8.12 | ESI^+^ |
| Eriocitrin | 596.53 | 595.10→287.07 | 40 | 18 | 9.41 | ESI^-^ |
| Liquiritin | 418.40 | 417.11→255.10 | 15 | 20 | 11.18 | ESI^-^ |
| Naringenin | 272.25 | 271.08→151.04 | 15 | 12 | 11.88 | ESI^-^ |
| Naringin | 580.53 | 579.13→271.09 | 30 | 32 | 13.81 | ESI^-^ |
| Hesperidin | 610.56 | 609.13→301.09 | 30 | 25 | 14.31 | ESI^-^ |
| Neohesperidin | 610.56 | 609.18→301.07 | 30 | 35 | 14.98 | ESI^-^ |
| Glycyrrhizic acid | 822.94 | 821.28→351.07 | 30 | 42 | 21.79 | ESI^-^ |
| Tangeretin | 372.37 | 373.13→343.08 | 5 | 20 | 24.28 | ESI^+^ |

**Table S2 Calibration curves, correlation coefficient (*r*) and linear ranges of 11 analytes.**

| Peak | Component | Calibration curves | *r* | Linear range (μg/mL) | LOQ (ng/mL) |
| --- | --- | --- | --- | --- | --- |
| 1 | Adenosine | *y =* 2,613,893.54 *x +* 158.07 | 0.9984 | 0.125-12.000 | 25.500 |
| 3 | Synephrine | *y =* 5,469,168.02 *x +* 114,540.94 | 0.9925 | 5.280-200.000 | 0.634 |
| 27 | Liquirtigenin | *y =* 375,555.87 *x -* 127.80 | 0.9987 | 1.300-16.280 | 450.000 |
| 31 | Eriocitrin | *y =* 21,019,939.13 *x -* 17,950.87 | 0.9983 | 2.960-12.410 | 14.400 |
| 37 | Liquiritin | *y =* 2,092,155.16 *x -* 3,276.22 | 0.9966 | 8.750-350.000 | 0.078 |
| 40 | Naringenin | *y =* 1,745,930.99 *x -* 9.98 | 0.9989 | 0.016-1.600 | 4.800 |
| 47 | Naringin | *y =* 314,451.12 *x +* 40,024.77 | 0.9928 | 47.000-1860.000 | 0.056 |
| 50 | Hesperidin | *y =* 2,629,600.53 *x +* 8,933.71 | 0.9970 | 1.000-240.000 | 0.120 |
| 60 | Neohesperidin | *y =* 987,177.61 *x +* 58,618.32 | 0.9994 | 3.000-1200.000 | 0.001 |
| 96 | Glycyrrhizic acid | *y =* 1,169,057.07 *x +* 27,315.20 | 0.9969 | 60.000-420.000 | 0.002 |
| 106 | Tangeretin | *y =* 24,743,287.99 *x +* 1,241.25 | 0.9984 | 0.043-2.000 | 3.600 |

**Table S3 Precision, repeatability and stability of 11 analytes (*n*=3).**

| Peak | Component | Precision (RSD, %) | Repeatability (RSD, %) | | Stability (RSD, %) | |
| --- | --- | --- | --- | --- | --- | --- |
| 1 | Adenosine | 6.04 | 8.17 | 6.47 | |  |
| 3 | Synephrine | 5.74 | 7.46 | 9.73 | |  |
| 27 | Liquirtigenin | 7.18 | 9.96 | 8.68 | |  |
| 31 | Eriocitrin | 8.16 | 8.93 | 8.90 | |  |
| 37 | Liquiritin | 9.56 | 4.65 | 9.89 | |  |
| 40 | Naringenin | 6.88 | 9.97 | 8.91 | |  |
| 47 | Naringin | 7.31 | 5.31 | 5.44 | |  |
| 50 | Hesperidin | 7.86 | 4.92 | 8.91 | |  |
| 60 | Neohesperidin | 4.49 | 4.70 | 7.23 | |  |
| 96 | Glycyrrhizic acid | 9.08 | 6.96 | 7.39 | |  |
| 106 | Tangeretin | 4.80 | 9.84 | 9.54 | |  |

**Table S4 Recoveries of 11 representative components in the WDD (*n*=3).**

| Peak | Component | Original (μg) | Spiked (μg) | Detected (μg) | Recovery (%) | RSD (%) |
| --- | --- | --- | --- | --- | --- | --- |
| 1 | Adenosine | 17.89 | 16.00 | 35.18±9.00 | 109.02±3.86 | 3.55 |
| 3 | Synephrine | 776.04 | 740.00 | 1543.99±0.32 | 103.56±4.90 | 4.73 |
| 27 | Liquirtigenin | 22.36 | 20.00 | 44.68±7.51 | 111.15±9.84 | 8.86 |
| 31 | Eriocitrin | 6.36 | 5.83 | 12.77±0.65 | 109.93±3.30 | 3.00 |
| 37 | Liquiritin | 1032.50 | 983.33 | 2025.62±0.31 | 100.78±3.71 | 3.68 |
| 40 | Naringenin | 1.87 | 1.90 | 3.75±0.21 | 98.82±5.49 | 5.55 |
| 47 | Naringin | 12343.93 | 12500.00 | 24653.31±0.66 | 98.85±6.21 | 6.29 |
| 50 | Hesperidin | 1122.82 | 1000.00 | 2200.87±0.18 | 107.80±7.19 | 6.67 |
| 60 | Neohesperidin | 3600.30 | 3500.00 | 7008.80±0.15 | 97.39±1.92 | 1.97 |
| 96 | Glycyrrhizic acid | 1331.61 | 1200.00 | 2593.75±0.19 | 105.18±7.09 | 6.74 |
| 106 | Tangeretin | 2.43 | 2.40 | 4.87±1.38 | 103.34±6.81 | 6.59 |

1. ^*^ Corresponding author.

   ^**^ Corresponding author.

   *E-mail addresses*: naturecolor@163.com (H.M. Zhang), xuhongxi88@gmail.com (H.X. Xu). [↑](#footnote-ref-2)
